# Phytochemical and comparative transcriptome analyses reveal different regulatory mechanisms in the terpenoid biosynthesis pathways between *Matricaria recutita* L. and *Chamaemelum nobile* L*.*

**DOI:** 10.1186/s12864-020-6579-z

**Published:** 2020-02-18

**Authors:** Yuling Tai, Xiaojuan Hou, Chun Liu, Jiameng Sun, Chunxiao Guo, Ling Su, Wei Jiang, Chengcheng Ling, Chengxiang Wang, Huanhuan Wang, Guifang Pan, Xiongyuan Si, Yi Yuan

**Affiliations:** 1 School of Life Science, Anhui Agricultural University, Hefei, 230036 China; 2 BGI Genomics, BGI-Shenzhen, Shenzhen, 518083 China

**Keywords:** Chamomile, Terpenoid biosynthesis, Essential oil, Comparative transcriptomics

## Abstract

**Background:**

*Matricaria recutita* (German chamomile) and *Chamaemelum nobile* (Roman chamomile) belong to the botanical family Asteraceae. These two herbs are not only morphologically distinguishable, but their secondary metabolites – especially the essential oils present in flowers are also different, especially the terpenoids. The aim of this project was to preliminarily identify regulatory mechanisms in the terpenoid biosynthetic pathways that differ between German and Roman chamomile by performing comparative transcriptomic and metabolomic analyses.

**Results:**

We determined the content of essential oils in disk florets and ray florets in these two chamomile species, and found that the terpenoid content in flowers of German chamomile is greater than that of Roman chamomile. In addition, a comparative RNA-seq analysis of German and Roman chamomile showed that 54% of genes shared > 75% sequence identity between the two species. In particular, more highly expressed DEGs (differentially expressed genes) and TF (transcription factor) genes, different regulation of CYPs (cytochrome P450 enzymes), and rapid evolution of downstream genes in the terpenoid biosynthetic pathway of German chamomile could be the main reasons to explain the differences in the types and levels of terpenoid compounds in these two species. In addition, a phylogenetic tree constructed from single copy genes showed that German chamomile and Roman chamomile are closely related to *Chrysanthemum nankingense*.

**Conclusion:**

This work provides the first insights into terpenoid biosynthesis in two species of chamomile. The candidate unigenes related to terpenoid biosynthesis will be important in molecular breeding approaches to modulate the essential oil composition of *Matricaria recutita* and *Chamaemelum nobile*.

## Background

German chamomile and Roman chamomile are the two most popular chamomile species in the Asteraceae, and the chemical compositions differ between the two species. The main characteristic constituents of chamomile are the essential oils in the flowers. Chamomile flowers are organized into flower heads that consist of a ring of male sterile outer ray florets (RF) and inner hermaphroditic disk florets (DF).

German chamomile is an annual herb native to Europe that has been known to humans as a medicinal herb and valued for thousands of years [1] for the characteristics of its aromatic oils. The flowers of German chamomile contain 0.2 to 1.9% essential oils [2] that consist mainly of terpenoids. As a traditional herbal medicine, German chamomile is widely used for the treatment of influenza, rheumatism pain, muscle spasm, gastrointestinal disorders, menstrual cramps, hemorrhoids, skin inflammation, and mucosal ulceration [3]. In addition, German chamomile has a mild calming effect, and can be used to reduce anxiety, treat convulsions, and as a sleep aid. Furthermore, chamomile is consumed as a popular herbal tea, and chamomile tea bags [4], which contain powdered chamomile flowers, are readily available on the market. In contrast to German chamomile, Roman chamomile is a perennial herb. The essential oil, which is present in the dried flowers of Roman chamomile (*Chamaemelum nobile*) at 0.3–1.5%, consists mainly of esters and a small amount sesquiterpenes such as angelic acid, angelic acid butyl ester, and chamazulene [5, 6]. While the essential oil is used mainly in cosmetics and perfumes, the primary medicinal uses are as a sedative, anxiolytic, and antispasmodic. The oil is also used to treat mild skin irritation and inflammation [3] [7, 8].

Terpenoids represent the largest class of floral volatiles and include such well known and widely distributed constituents of floral scents as the monoterpenes linalool, limonene, myrcene, ocimene, and geraniol, and the sesquiterpenes farnesene, nerolidol, caryophyllene, and germacrene. Generally, there are two well-established pathways generating IPP (isopentenyl pyrophosphate) and DMAPP (dimethylallyl diphosphate) in plants: one is the mevalonate (MVA) pathway and the other is the methylerythritol phosphate (MEP) pathway [9]. In recent studies, monoterpene synthases have been identified in plastids and may also be present in the cytosol [10, 11] and several studies have indicated that molecules such as GPP can be exported from the plastids to the cytosol [12]. In addition, sesquiterpenes were first thought to be synthesized exclusively in the cytosol, using precursors generated via the MVA pathway. However, there seem to be some exceptions to this rule, such as snapdragon flowers [13] and tea [14]. *Haematococcus pluvialis*, a species of green alga, differs from green plants in that it may synthesize isoprenoids exclusively via the MEP pathway [15]. Some diterpenes are also made in the cytosol [16]. All of this indicates that these two pathways (MVA and MEP) are not independent, and cross-talk between them has also been documented. Because the main terpenoids present in German chamomile and Roman chamomile flowers are mainly monoterpenes and sesquiterpenes, the particular pathway from which the precursors are derived is unknown. Our study analyzed the transcription of key genes in these two pathways, and deduced the possible synthesis by way of isopentenyl pyrophosphate (IPP). We performed RNA-seq analyses on disk and ray florets of both German chamomile and Roman chamomile to examine differences in gene expression between the two chamomile species. This comparative transcriptomic analysis provides important insights into the molecular mechanisms that regulate the terpenoid biosynthesis pathways.

Transcriptomic analyses can be used to identify functional elements in the genome, metabolic pathways, and differentially expressed genes in model and non-model organisms [17–20]. The transcriptomes of many important medicinal plants and their individual tissues such as leaves, roots, and stems have been reported [21–25]. Genomic resources and transcriptome sequences for German chamomile and Roman chamomile are very limited at present, and the complex regulatory mechanisms that control carbon flux through the terpenoid biosynthetic pathway and their cooperation in the biosynthesis of volatile terpenoids remain unknown. To facilitate further research on the biosynthesis of secondary metabolites, we focused on the terpenoid metabolic pathways in German and Roman chamomile, including the regulatory relationships between genes for key enzymes and transcription factors. In the current study, we compared the disk floret and ray floret transcriptomes of German chamomile and Roman chamomile using RNA-seq analyses, and identified genes related to terpenoid biosynthesis. This is the first report of the application of RNA-seq to German and Roman chamomile. The data generated in this study will be a useful resource for future genetic and genomic studies in *M. recutita* and *C. nobile*.

## Results

### Determination of essential oil constituents in flowers of German and Roman chamomile

**Fig. 1:**
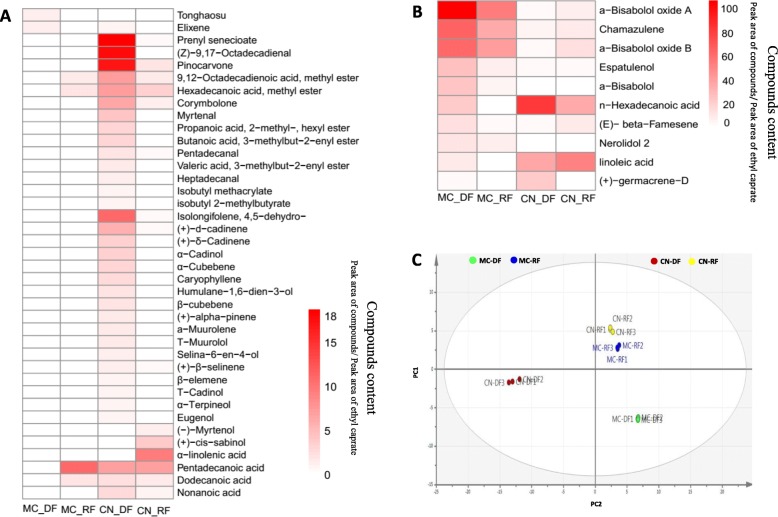
Heat map of essential oil content (**a** and **b**) and principal component analysis (OPLS-DA) plot (**c**) of data from essential oil of two different kind flowers from German Chamomile (MC) and Roman chamomile (CN) detection by GC-MS. MC_DF: Disk florets of German chamomile; MC_RF: Ray florets of German chamomile; CN_DF: Disk florets of Roman chamomile; CN_RF: Ray florets of Roman chamomile. The scale is a relative scale (peak area of compounds/ peak area of ethyl caprate), and the used ethyl caprate was used as internal standard to calculate the relative content of essential oil components in German and Roman chamomile.

**Fig. 2:**
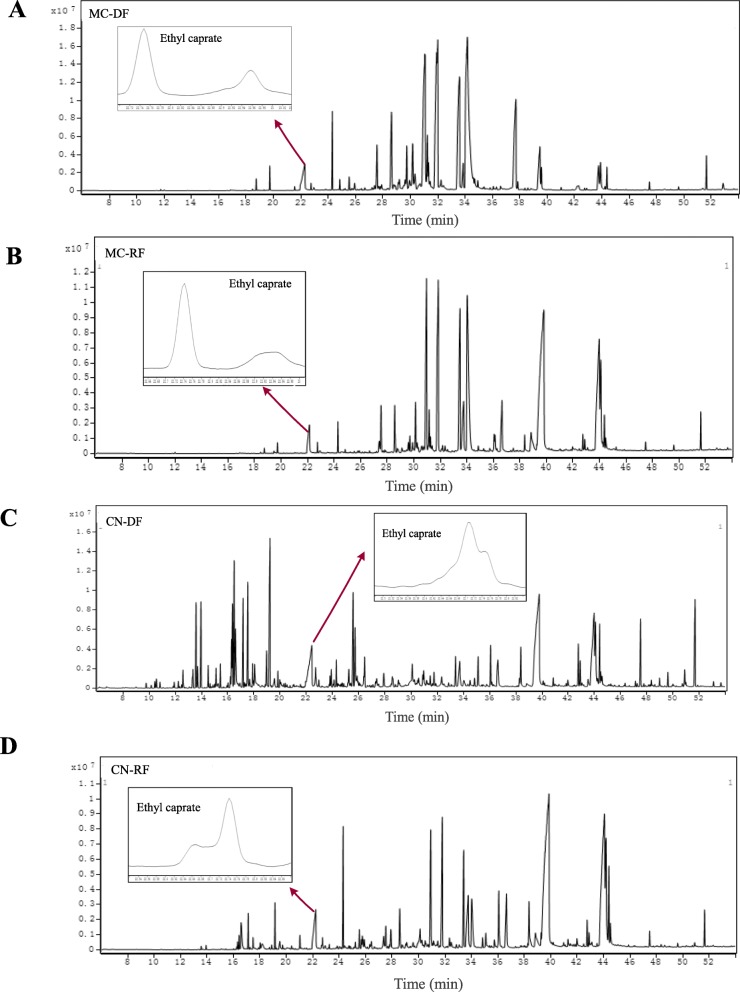
Total Ion Chromatorgraphy of essential oil from disk florets of German chamomile (**a**), ray florets of German chamomile (**b**), disk florets of Roman chamomile (**c**) and ray florets of Roman chamomile (**d**). Ethyl caprate: the internal standard for calculating relative peak ratios

GC-MS analysis indicated that the essential monoterpenoid and sesquiterpenoid constituents in the flowers of German chamomile and Roman chamomile are significantly different (Fig. 1 and Fig. 2, Additional file 1). The relative quantities of monoterpenoids and sesquiterpenoids in disk flowers were always higher than in ray flowers in both chamomile species. The main compounds present in German chamomile essential oil are sesquiterpenoids, such as α-Bisabolol oxide A, Chamazulene, α-Bisabolol oxide B, α-Bisabolol, and Espatulenol. The major constituents in Roman chamomile essential oil are n-Hexadecanoic acid, linoleic acid, and other esters. The levels of α-Bisabolol oxide A, α-Bisabolol oxide B, and Chamazulene in German chamomile were 50-, 30-, and 10-fold higher than in Roman chamomile. In addition, the contents of these compounds in disk flowers were 2 to 10-fold greater than in the ray flowers of the two chamomiles. These results are consistent with previous findings reported by Yao et al. [26].

### De novo transcriptome assembly and comparative RNA-seq analyses

After removing the terminal adaptor sequences, duplicated and ambiguous sequences, and low-quality reads, we generated approximately 106.72 Gb of Illumina RNA-seq data from mRNA extracted from ray flowers and disk flowers of German and Roman chamomile. We used 53.31 Gb and 53.41 Gb of clean read data in the transcriptome assemblies for German and Roman chamomile, respectively. The Q20 and Q30 scores were greater than 98 and 95%, respectively, for the German and Roman chamomile transcriptome data. The final German chamomile cDNA assembly consisted of 117,203 unigenes; the average length was 1056 bp and the N50 length was 1686 bp. The final assembly for Roman chamomile consisted of 147,616 unigenes with an average length of 914 bp and an N50 length of 1506 bp (Table 1). There were 50,881 (43.41%) and 48,957 (33.17%) unigenes >1000 bp in length in German and Roman chamomile, respectively.

**Table 1:** Summary of RNA-seq and unigene data from German and Roman chamomile

	German chamomile	Roman chamomile
Total Raw Reads (Gb)	55.9	56.3
Total Clean Bases (Gb)	53.31	53.41
Clean Reads Q20 (%)	> 98%	>98%
Clean Reads Q30 (%)	> 95%	>95%
Average unigene length	1056	914
Total number of unigenes	117,203	147,616
N50 value	1686	1506
GC (%)	40.12	40.86

### Functional annotation and classification

All unigenes from both German chamomile and Roman chamomile were annotated using several public databases: Nr (NCBI non-redundant protein sequences), Nt (non-nucleotide), Swiss-Prot, COG (Clusters of Orthologous Groups of proteins), KEGG (the Kyoto Encyclopedia of Genes and Genomes), and GO (Gene Ontology). There were 89,796 (60.83%) and 73,699 (62.88%) sequences annotated in the German chamomile and Roman chamomile transcriptomes, respectively. We annotated 68,325 (NR: 58.30%), 47,469 (NT: 40.50%), 48,902 (Swissprot: 41.72%), 29,222 (COG: 24.93%), 52,423 (KEGG: 44.73%), 11,385 (GO: 9.71%), and 51,613 (Interpro: 44.04%) unigenes in the Roman chamomile transcriptome. Similarly, 80,594 (NR: 54.60%), 49,537 (NT: 33.56%), 56,793 (Swissprot: 38.47%), 35,889 (COG: 24.31%), 62,060 (KEGG: 42.04%), 13,766 (GO: 9.33%), and 63,945 (Interpro: 43.32%) unigenes were annotated in the German chamomile transcriptome using the seven functional databases (Table 2).

**Table 2:** Functional annotation of floret unigenes in German and Roman chamomile

Values	German chamomile		Roman chamomile	
	Number	Percentage	Number	Percentage
Total	147,616	100%	117,203	100%
Nr-Annotated	80,594	54.60%	68,325	58.30%
Nt-Annotated	49,537	33.56%	47,469	40.50%
Swissprot-Annotated	56,793	38.47%	48,902	41.72%
KEGG-Annotated	62,060	42.04%	52,423	44.73%
COG-Annotated	35,889	24.31%	29,222	24.93%
Interpro-Annotated	63,945	43.32%	51,613	44.04%
GO-Annotated	13,766	9.33%	11,385	9.71%
Overall	89,796	60.83%	73,699	62.88%

Overall, 29,222 (24.93%) unigenes in the German chamomile transcriptome were assigned to 25 COG categories (Supplementary Figure 1A). Among these groups, unigenes belonging to “general function prediction” occupied the largest part (8163), followed by “transcription” (4382). In addition, 35,889 of the total 117,203 Roman chamomile unigenes were classified into 25 COG categories. The assignments (10,007) were mostly enriched in the “general function prediction”, followed by the “transcription” clusters (5192) (Supplementary Figure 1B). KEGG pathway analysis was performed to further predict gene function in the biological pathways of the assembled unigenes in the German chamomile and Roman chamomile transcriptomes. In total, 62,060 unigenes in German chamomile were assigned to 135 signal pathways. Among these, 1520 unigenes were annotated in “Metabolism of terpenoids and polyketides”. Also, 52,423 unigenes in Roman chamomile were categorized into 136 pathways, and 1633 unigenes were annotated in “Metabolism of terpenoids and polyketides” (Supplementary Figure 2).

### Identification of DEGs and further analysis of the terpenoid biosynthesis pathway

**Fig. 3:**
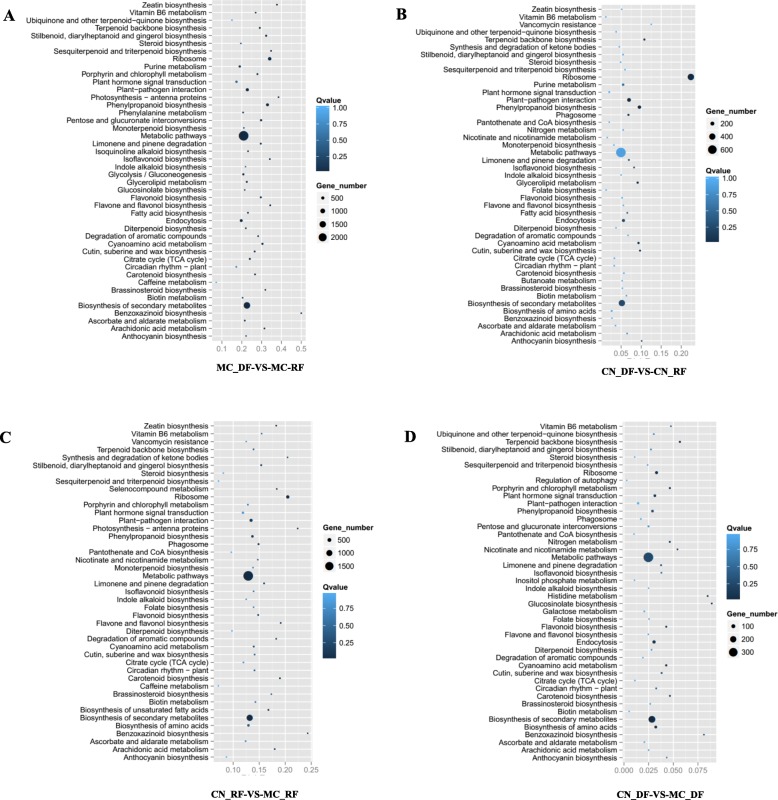
Pathway enrichment analysis by tissue pair comparisons. The ratio between the number of DEGs mapped to a pathway and the total number of genes mapped to that pathway are indicated by enrichment factors. **a** Disk florets of German chamomile vs. Ray florets of German chamomile (MC_DF-VS-MC-RF), **b** Disk florets of Roman chamomile vs. Ray florets of Roman chamomile (CN_DF-VS-CN_RF), **c** Ray florets of Roman chamomile vs. Ray florets of German chamomile (CN_RF-VS-MC_RF), **d** Disk florets of Roman chamomile vs. Disk florets of German chamomile CN_DF-VS-MC_DF. A larger enrichment factor indicates greater intensiveness. X-axis represents KEGG pathways, Y-axis represents enrichment factors. The Q values were calculated using a hypergeometric test with the Bonferroni Correction. The Q value is a corrected *p* value that ranges from 0 to 1, and lower Q values mean greater intensiveness. “Gene number” refers to the number of DEGs mapped to a given pathway.

We identified DEGs by comparing the FPKM (Fragment Per Kilobase of exon model per Million mapped reads) values between the different libraries; thresholds were log_2_ fold-change > 1 and the FDR (False Discovery Rate) was ≤0.001 [27]. We used the number of DEGs mapping to a pathway/total number of genes mapped to the pathway (enrichment factors) to estimate the relative degree of enrichment in these pathways. The maximum enrichment factor (0.5) for pathways in the MC_DF (German chamomile disk florets) vs. MC_RF (German chamomile ray florets) comparison was benzoxazinoid biosynthesis, followed by zeatin biosynthesis (0.38), sesquiterpenoid and triterpenoid biosynthesis (0.35), and flavone and flavonol biosynthesis (0.34). Also, the enrichment factors for terpenoid backbone biosynthesis and diterpenoid biosynthesis were 0.29 and 0.22, respectively. In the CN_DF (Roman chamomile disk florets) vs. CN_RF (Roman chamomile ray florets) comparison, the top three were the ribosome (0.22), vancomycin resistance (0.12), and terpenoid backbone biosynthesis (0.11). In CN_RF vs. MC_RF the top three were benzoxazinoid biosynthesis (0.24), histidine metabolism (0.23), and photosynthesis - antenna proteins (0.22); in addition, carotenoid biosynthesis was 0.19 and limonene and pinene degradation was 0.15. In CN_DF vs. MC_DF, the top three were glucosinolate biosynthesis (0.089), histidine metabolism (0.084), and benzoxazinoid biosynthesis (0.081); terpenoid backbone biosynthesis was 0.057 (Fig. 3 and Additional file 2). DEGs identified by comparing RF with DF clustered in the pathways for disease and pest resistance and terpenoid metabolism. The DEGs between German chamomile and Roman chamomile were clustered in disease and pest resistance pathways. The enrichment factors in the MC_DF vs. MC_RF comparison were higher than in the CN_DF vs. CN_RF comparison, and the enrichment factors in CN_RF vs. MC_RF were higher than in CN_DF vs. MC_DF.

**Fig. 4:**
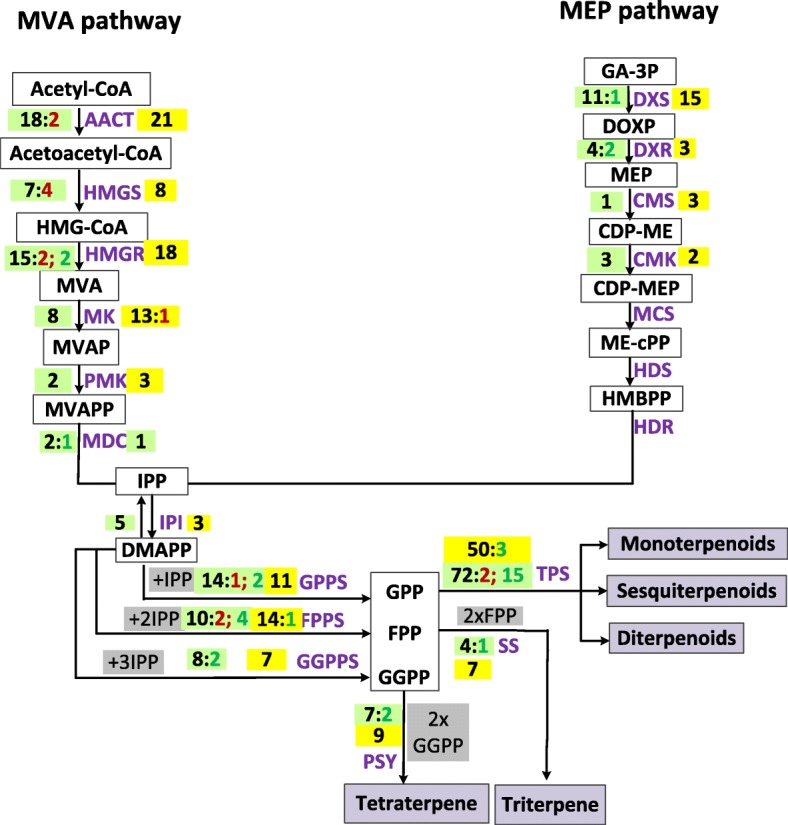
Number of annotated genes and DEGs related to terpenoid biosynthetic pathway in German chamomile (green) and Roman chamomile (yellow) Compound names are shown below each arrow. Abbreviations beside the arrows indicate the enzymes catalyzing the reaction. The numbers written in black indicate the total number of genes in this pathway; numbers in red show the number of up-regulated (green: MC_DF vs MC-RF; yellow CN_DF vs CN -RF) genes, and those in green show the number of down-regulated (green: MC_DF vs MC-RF; yellow CN_DF vs CN -RF) genes

We also identifed DEGs in the terpenoid biosynthetic pathways of German and Roman chamomile. A schematic representation of the DEGs and annotated genes in the biosynthetic pathways for these compounds is shown in Fig. 4 [28]. Terpenoid biosynthesis utilizes isoprenoid precursors from terpenoid backbone biosynthesis (MVA and MEP pathways). In the MVA pathway, two AACT, four HMGS, and two HMGR were up regulated in MC-DF vs. MC-RF. In the MEP pathway, one DXS (1-deoxy-D-xylulose-5-phosphate synthase) and two DXR (1-deoxy-D-xylulose-5-phosphate reductoisomerase) genes were down regulated in MC-DF vs MC-RF. Also, DXS and DXR may play rate-limiting roles in controlling metabolic flux through the MEP pathway [29]. A previous study reports that DXS and HDR are both encoded by small gene families in higher plants, and influence the accumulation of downstream isoprenoids [30]. For example, the three DXS genes in maize (*Zea mays*) encode functional enzymes, and two different HDR genes have been identified in loblolly pine (*Pinus taeda*) [31, 32].

In the MC-DF vs. MC-RF comparison, AACT and HMGS are both up-regulated in the terpenoid biosynthesis pathway. There are two and four down-regulated DEGs related to GPPS (geranyl pyrophosphate synthase) and FPPS (farnesyl pyrophosphate synthase), respectively, and DEGs related to GGPPS (geranylgeranyl pyrophosphate synthase), SS (squalene synthase), and PSY (phytoene synthase) were all up-regulated. Also in MC-DF vs MC-RF, 17 DEGs related to terpene synthase (TPS) were identified, and among them, the relative expression of 15 TPS genes was down-regulated. Terpenoid biosynthesis in plants is catalyzed by a family of enzymes known as terpene synthases that convert prenyl diphosphates to various subclasses of terpeneoids [33]. In the CN-DF vs. CN-RF comparison, there were one and three DEGs related to FPPS and TPS that were down-regulated in expression. These results indicate that the gene expression levels of the rate-limiting upstream enzymes and a variety of TPS genes downstream could result in the observed differences in both the variety and contents of terpenoids in flowers of German and Roman chamomile.

TFs play a diverse role in regulating secondary metabolism pathways by turning genes on and off in plants [34]. We searched for candidate TF genes in the transcriptomes of German chamomile and Roman chamomile, and identified 94 differentially expressed transcription factor genes (52 up-regulated and 42 down-regulated) in CN_DF vs. CN_RF. We also identified 59 differentially expressed (31 and 28 up- and down-regulated, respectively) transcription factor genes in CN_DF vs. MC_DF, 328 (167 up-regulated and 161 down-regulated) in CN_RF vs. MC_RF, and 479 (267 up-regulated and 212 down-regulated) in the in MC_DF vs. MC_RF comparison (Additional file 3).

### Construction and analysis of a protein–protein interaction network (PPIN) between German chamomile and Roman chamomile

**Fig. 5:**
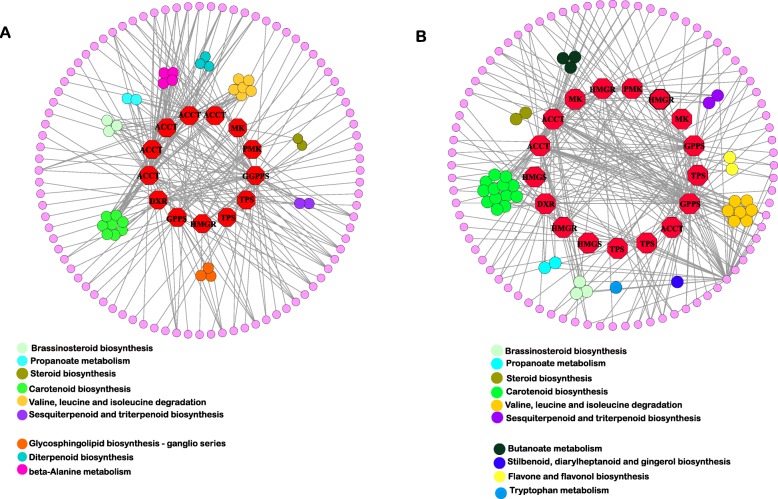
PPIN involved in terpenoid biosynthetic pathway in German chamomile (**a**) and Roman chamomile (**b**) DEGs between German and Roman chamomile (query coverage > = 50% and identity > = 40%) were identified as the homology interacting protein, and were built a protein interaction network. Octagon with red color represents genes involved in terpenoid biosynthetic pathway. Circle represents genes which were interacted with genes involved in terpenoid biosynthetic pathway. Circle with different color represents genes involved in different KEGG pathways.

A total of 477 and 505 interacting pairs involved in terpenoid biosynthesis were identified from German chamomile and Roman chamomile, respectively. We selected the interacting proteins for further analysis, and the PPIN related to the terpenoid biosynthesis pathway is shown in Fig. 5. We found that these proteins are involved in “sesquiterpenoid and triterpenoid biosynthesis”, “brassinosteroid biosynthesis”, “propanoate metabolism”, “steroid biosynthesis”, “carotenoid biosynthesis” and “valine, leucine and isoleucine degradation” in German chamomile and Roman chamomile. In addition, we also identified three, three, and four proteins involved in “glycosphingolipid biosynthesis - ganglio series”, “diterpenoid biosynthesis”, and “beta-alanine metabolism” in German chamomile. In Roman chamomile, we identified three, one, two, and one proteins involved in “butanoate metabolism”, “stilbenoid, diarylheptanoid and gingerol biosynthesis”, “flavone and flavonol biosynthesis” and “tryptophan metabolism”.

We found a number of CYPs (cytochrome P450 enzymes) that interact with proteins involved in terpenoid biosynthesis in the PPINs for German chamomile and Roman chamomile. There were more types and more CYP-protein interactions in German chamomile than in Roman chamomile. In addition, we found transcription factors such as MYB, WD-40 repeat, and zinc finger proteins in the PPINs of German chamomile and Roman chamomile, and the types and number of interactions were also different (Fig. 5 and Additional file 4).

### Comparison of nucleotide sequence identity and divergence analysis between German and Roman chamomile

**Fig. 6:**
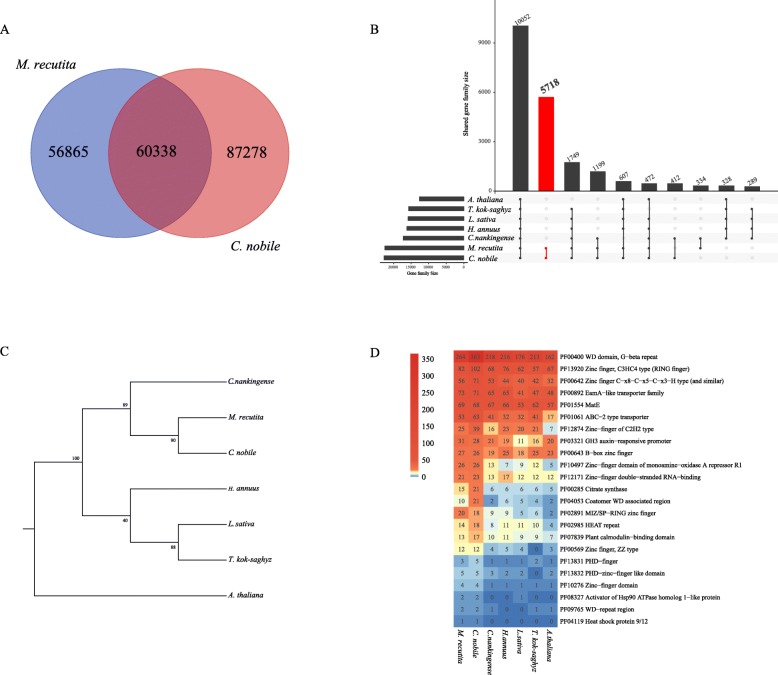
Comparison analysis in German chamomile, Roman chamomile and other studied species (**a**) Unigenes comparison between German chamomile and Roman chamomile. Protein sequences from German and Roman chamomile were compared, > 80% of the length of each gene in a pair of homologous genes was strictly aligned, and sequences with > 70% homology were identified as homologous genes. The remaining sequences were identified as special transcripts in the German and Roman chamomile transcriptomes. **b** Analysis of gene families in two chamomiles and other studied species. The histogram on the left represents the number of gene families identified for each species, and the histogram above shows the number of common gene families among line connected species. Red color represents gene families specially presented in German and Roman chamomile. **c** Phylogenetic tree of two chamomiles and other studied species based on single-copy genes. The phylogenetic tree was constructed by FastTree (2.1.10) and the number on the node represents the bootstrap value. **d** Pfam domain enrichment analysis of genes families in German chamomile, Roman chamomile and other studied species using Fisher’s exact test. The color represents gene numbers in each species

A high level of nucleotide sequence similarity was found in genes that are homologous between German and Roman chamomile, with 54% of the genes sharing > 75% identity. We obtained 60,338 transcripts that were common to German Chamomile and Roman chamomile. We also found 56,865 and 87,278 transcripts that are specific to German and Roman chamomile (special transcripts), respectively (Fig. 6a). In addition, the special transcripts identified in the two species were used as queries to search the KEGG database; we found that 2960 out of 6811 (43%) and 3861 out of 7772 (49%) of the unigenes are predicted to be involved in secondary metabolite biosynthesis in German and Roman chamomile, respectively. Also, 1000 unigenes out of 2194 (46%) 1295 out of 2588 (50%) were related to plant-pathogen interactions in German and Roman chamomile, respectively (Additional file 5). A total of 27,886 putative ortholog pairs were identified between German and Roman chamomile. Of these ortholog pairs, 584 had Ka/Ks values > 1, suggesting that they are under positive selection. Among these positive selection genes, there was one unigene (CMK: 4-diphosphocytidyl-2-C-methyl-D-erythritol kinase) related to terpenoid backbone biosynthesis, one unigene (germacrene D synthase) related to sesquiterpenoid biosynthesis, two unigenes ((E)-beta-ocimene synthase and (+)-neomenthol dehydrogenase) related to monoterpenoid biosynthesis and one unigene (*Artemisia annua* cytochrome P450 mono-oxygenase) related to diterpenoid biosynthesis (Table 3). Since the Ka/Ks ratio is widely used to detect selective pressure (environmental factors) acting on protein-coding sequences, rapid evolution of the genes involved in terpenoid backbone biosynthesis, monoterpenoid biosynthesis, sesquiterpenoid biosynthesis, and diterpenoid biosynthesis could be associated with adaptive selection between German and Roman chamomile.

**Table 3:** Genes related to the terpenoid biosynthesis pathway with Ka/Ks >1

Pathway	GenePair (MC)	GenePair (CN)	Ka	Ks	ka/ks	Description
Terpenoid backbone biosynthesis	CL6716.Contig2	Unigene23705	0.0094	0.0088	1.0681	4-diphosphocytidyl-2-C-methyl-D-erythritol kinase
Monoterpenoid biosynthesis	Unigene31629	Unigene603	0.0093	0.0085	1.0941	(E)-beta-ocimene synthase
Sesquiterpenoid biosynthesis	Unigene29935	CL9420.Contig2	0.0122	0.0034	3.5882	Germacrene D synthase
Monoterpenoid biosynthesis	CL6029.Contig1	CL15218.Contig2	0.0059	0.0049	1.204	(+)-neomenthol dehydrogenase
Limonene and pinene degradation	CL15540.Contig9	CL3089.Contig1	0.0082	0.0047	1.7446	Parthenolide synthase
Limonene and pinene degradation	Unigene10231	CL15190.Contig1	0.0054	0.0049	1.102	Aldehyde dehydrogenase
Diterpenoid biosynthesis	CL14129.Contig2	CL8947.Contig2	0.0037	0.0014	2.6428	*Artemisia annua* cytochrome P450 mono-oxygenase

### Comparative transcriptomic analysis of German chamomile, Roman chamomile, and other studied species

A total of 92,393 gene families were identified by orthofinder, among which there were 1232 single-copy genes. There were six gene families specific to German chamomile, and eight gene families were specific to Roman chamomile. In the German chamomile-specific gene families, we identified one gene family related to disease resistance. A total of 5718 gene families were found specifically in both German chamomile and Roman chamomile (Fig. 6b). The KEGG enrichment results showed that gene families specific to both German chamomile and Roman chamomile were mainly enriched in ‘metabolism’, and 472 gene families were enriched in ‘biosynthesis of secondary metabolites’ (Additional file 6). The phylogenetic tree constructed from single-copy gene sequences shows that German chamomile and Roman chamomile are in a clade with *Chrysanthemum nankingense* (Fig. 6c). In addition, we analyzed unigenes from German chamomile, Roman chamomile and other studied plants based on the Pfam database, and the results showed that unigenes encoding TFs such as WD-repeat and zinc-finger proteins were more prevalent in German chamomile and Roman chamomile than in other studied specie. Furthermore, there were more MATE and ABC transporter genes in German chamomile and Roman chamomile (Fig. 6d).

### qRT-PCR analysis and correlation analysis

We used qRT-PCR (quantitative real-time PCR) analyses to examine the expression levels of 21 DEGs from the transcriptomic libraries of German chamomile (14 DEGs) and Roman chamomile (7 DEGs). Of these genes, 19 showed differential expression levels that matched the RNA-seq data. The agreement rate was 90.48% (Supplementary Figure 3 and Additional file 7). These results verified the accuracy of the transcriptome data. The three replicate samples from both German chamomile and Roman chamomile showed good correlations; the correlation index was > 0.84 for CN-DF-1, CN-DF-2 and CN-DF-3, and it was > 0.99 for CN-RF-1, CN-RF-2, and CN-RF-3; MC-DF-1, MC-DF-2 and MC-DF-3; and MC-RF-1, MC-RF-2 and MC-RF-3, which indicated that the transcriptome data was relatively accurate. However, the correlation index between MC-DF and CN-DF was ~ 0.8, and the correlation index between MC-RF and CN-RF was ~ 0.85. The correlation index between MC-DF and MC-RF was only 0.48–0.49, and it was 0.5–0.63 between CN-DF and CN-RF (Supplementary Figure 4).

## Discussion

Of the many species of chamomile, two of the most popular are German and Roman chamomile, and both species are in the Asteraceae family. German chamomile is more widely grown than Roman chamomile [35, 36]. The medicinal value of German chamomile is related to the content of the essential oil that is mainly produced in the flowers. The main components of the essential oil are monoterpenoids and sesquiterpenoids, such as (E)-β-farnesene, terpene alcohol, chamazulene, α-bisabolol (4.8–11.3%), and α-bisabolol oxides A and B. The levels of α-bisabolol and α-bisabolol oxides A and B in the flowers peak at full bloom and then decline [37]. The composition of essential oils is quite different between Roman and German chamomile. As discussed above, the essential oil of German chamomile is composed mainly of sesquiterpenoids such as chamazulene, bisabolol, and its oxide, while the major constituents of Roman chamomile oil are angelates, such as angelic acid and its esters, and monoterpenoids such as α-pinene [38] [39]. In this study, we analyzed and compared the differences in the compounds between the two species of chamomile. Our results showed that there were more kinds of monoterpenoids and sesquiterpenoids in German Chamomile than in Roman Chamomile, and that the content was higher. In addition, there were more kinds monoterpenoids and sesquiterpenoids, and the content was also higher, in disk florets compared to ray florets.

There are at least two ways in which the biosynthesis of terpenoids can be regulated: (1) at the level of terpenoid backbone biosynthesis and (2) at the level of substrates for TPSs. Previous studies have suggested that in the terpenoid backbone biosynthesis pathway, the rate of metabolic flux through the MEP pathway is controlled by DXS and DXR [29]. Furthermore, DXS and HDR are encoded by small gene families; there are three functional DXS genes in maize [31] and two different HDR genes in *Pinus taeda* [32]. In this study, we found 11 DXS and 4 DXR genes in the MC-DF vs MC-RF comparison. Also, two genes encoding AACTs, two genes encoding HMGRs and four genes encoding HMGSs were found to be up-regulated. We therefore speculated that increased gene expression in the MVA pathway could provide more substrate for the downstream pathway. The relative level of gene expression of the key genes (such as gene encoding DXS, DXR, MDC, SS, and PSY) is higher in MC-DF vs. MC-RF. We also found that the expression levels of genes encoding FPS and TPS was higher in CN-DF vs. CN-RF. While TPS gene regulation has previously been shown to be important [40], our results indicate that up-regulated TPSs and higher expression levels of key genes in the disk florets might lead to the higher contents of monoterpenoids and sesquiterpenoids found in German chamomile. We also identified seven genes under accelerated evolution with Ka/Ks ratios > 1 in the terpenoid biosynthesis pathway. These rapidly-evolving genes may be the one reason for the differences observed between German and Roman Chamomile.

Transcription factors (TFs), which are generally DNA binding proteins, use a variety of mechanisms to regulate gene expression in response to changes in environmental conditions, during development, in response to stress, and in defense responses through interactions directed towards plant pathogens [41]. WRKY, NAC, DOF, MYB, the Zinc finger family, F-Box Homeobox, the WD40 repeat family, bHLH, pathogenesis-related/ERF, and AP2 are commonly-identified classes of TFs [42–44]. Spyropoulou et al. identified a pool of TFs involved in terpene synthesis using RNA sequencing in *Solanum lycopersicum* [45], and Suttipanta et al. found that in *Catharanthus roseus*, ORCA2 and an AP2 family member, MYC2, a bHLH family member, and WRKY1 together regulate indole alkaloid and terpenoid biosynthesis [46, 47]. In this study, we compared genes between German chamomile and Roman chamomile, as well as from other Compositae family plants based on Pfam database, and the results showed that unigenes encoding TFs such as WD-repeat and zinc-finger proteins were more prevalent in German chamomile and Roman chamomile compared to other plants in the Compositae. We also found that there are more genes for MATE and ABC transporters in German chamomile and Roman chamomile.

CYPs (cytochrome P450 enzymes) not only play indispensable roles in plant primary metabolism but are also important in secondary metabolism. Recent studies have shown that CYPs function as modification enzymes in terpenoid biosynthesis [48, 49]. In addition, we identified many CYPs that interact with proteins involved in terpenoid biosynthesis in PPINs for German chamomile and Roman chamomile, and we found that there are more CYP-interacting proteins in German chamomile compared to Roman chamomile. This result reinforced our finding that the terpenoid biosynthesis pathway in German chamomile is more active that in Roman chamomile, and higher expression levels of DEGs, and the regulation of TFs and CYPs could result in the observed differences in terpenoid content between German and Roman chamomile.

## Conclusions

Previous studies have shown that essential oil compositions vary in German chamomile as compared to Roman chamomile [26, 50]. In this study, we found there are more monoterpenoids and sesquiterpenoids in flowers of German chamomile compared to Roman chamomile, and these compounds were more prevalent in disk florets than in ray florets. Furthermore, we obtained a large number of unigenes by transcriptome sequencing in German and Roman chamomile. We identified many candidate unigenes related to terpenoid biosynthesis, and the majority were more highly expressed in MC-DF than in MC-RF, especially the key enzyme genes in this pathway. The DEGs, TFs, and CYPs involved in the terpenoid biosynthesis pathway were more numerous in German chamomile than in Roman chamomile. In addition, we identified genes related to terpenoid biosynthesis pathway that appear to be under accelerated evolution, which could explain the differences in the chemical compounds and metabolite biosynthesis between the two species. A phylogenetic tree based on single copy genes showed that German chamomile and Roman chamomile are closely related to *Chrysanthemum nankingense*. Our study is the first to report a comparative transcriptome analysis between German chamomile and Roman chamomile. The transcriptomic data generated in this study will be an invaluable resource for further studies involving functional genomics, molecular biology, and plant breeding in German chamomile and Roman chamomile.

## Methods

### Plant materials

Plants of German chamomile (*Matricaria recutita*) and Roman chamomile (*Chamaemelum nobile*) were obtained from YuePing Ltd. and were grown on the experimental farm at AnHui Agricultural University, HeFei, China. Full-bloom-stage flowers of German chamomile and Roman chamomile were collected from 6 month-old plants, and the disk and ray florets were separated immediately after collection (Supplementary Figure 5). All samples were identified by Prof. Yi Yuan. The essential oils were then extracted from the dried disk and ray florets of German chamomile and Roman chamomile. A portion of the disk and ray florets of German chamomile and Roman chamomile were flash frozen in liquid nitrogen and stored at − 80 °C to be used for RNA extraction. All samples consisted of three biological replicates that were collected from different plants.

### GC-MS (gas chromatography-mass spectrometry) analysis of the essential oils extracted from German and Roman chamomile flowers

The essential oils of German chamomile and Roman chamomile flowers were extracted using steam distillation. Dried samples (50 g) were crushed and were then refluxed for 8 h in a round bottom flask containing 1000 ml of pure water. We analyzed samples with three biological replicates. GC conditions: The analytes were separated using an HP-5MS column (30 m × 0.25 mm I.D. × 0.25 μm film thickness; Agilent Technologies, USA); the carrier gas was high-purity helium (99.999%, Airgas Inc.); the flow rate was 1 mL/min; the injector temperature was 280 °C; The injection mode was split, and the split ratio was 10:1. The oven temperature was maintained at 70 °C for 3 min, then programmed to rise from 70 °C to 180 °C at the rate of 5.5 °C/min, and was then held at 180 °C for 4 min. The temperature was programmed to rise to 280 °C at the rate of 4 °C/min, and was then held at 280 °C for 2 min. MS conditions: the transfer line temperature was 280 °C; ionization mode, electron impact (EI) at 70 eV; the quadrupole temperature was 150 °C; scan range, m/z 29–420; scan rate, 3.75 scans/s; with an ion source temperature of 230 °C. Compounds were identified by comparing the mass spectra and retention indices with spectra in the NIST database [51]. We used ethyl caprate as the internal standard to calculate relative peak ratios (peak area of compounds/ peak area of ethyl caprate) in German chamomile and Roman chamomile.

### RNA extraction, library construction, and RNA sequencing

Total RNA was extracted separately from flowers of German chamomile and Roman chamomile using the modified CTAB (cetyl trimethyl ammonium bromide) method with three biological replicates [52]. The yield and quality of RNA was determined using gel electrophoresis and spectrophotometry (Nanodrop 2000). Enrichment of mRNA, fragmentation, cDNA synthesis, adapter addition, fragment size selection, PCR amplification, and RNA-seq were performed at the Beijing Genome Institute (BGI; Shenzhen, China). As a final step, the cDNA libraries were examined using an Agilent 2100 Bioanaylzer and the ABI StepOnePlus Real-Time PCR System prior to sequencing on the Illumina HiSeq 4000.

### De novo assembly of chamomile flower transcriptomes

Clean reads from the 12 cDNA libraries were obtained by removing low quality reads, adaptor sequences, and reads with a high content of unknown bases (Ns) from the raw reads. De novo transcriptome assemblies for the four samples were performed separately using the transcriptome assembler Trinity [53], and each sample had three replicates. Unigenes were generated by removing the redundant sequences and short transcripts. The unigene ORFs were predicted and translated by TRANSDECODER (https://github.com/TransDecoder/TransDecoder/releases). The German chamomile and Roman chamomile unigenes were then aligned to each other by the RBH method described in Tai et al. [17].

### Functional annotation and classification of the unigenes

The unigenes were compared against the Nr, Swiss-Prot, COG [54], and Nt sequences databases to retrieve protein functional annotations using Blastx or Blastn with a threshold E-value of 1 × 10^− 5^. Metabolic pathway assignments for the unigenes were obtained using KEGG annotation [55] to understand the complex biological functions of the gene products. Orthologous gene products can be classified, and the potential functions of the unigenes predicted, using the COG database. Based on Nr searches, the GO classifications of the unigenes were obtained with WEGO software [56, 57] to show the distribution of gene functions in the “Cellular Component”, “Molecular Function”, and “Biological Process” GO domains.

### Comparison of nucleotide and protein sequences between German chamomile and Roman chamomile

Predicted protein sequences from the German and Roman chamomile flower transcriptomes were compared using BLAST and MUMmer (http://mummer.sourceforge.net/); > 80% of the length of each gene in a pair of homologous genes was strictly aligned, and sequences with > 70% homology were identified as homologous genes. The remaining sequences were identified as special transcripts in the German and Roman chamomile transcriptomes.

### DEGs related to major secondary metabolism pathways

The calculation of relative unigene expression uses the FPKM method (Fragment Per Kilobase of exon model per Million mapped reads) [27]. The identification of DEGs was performed based on “The significance of digital gene expression profiles” [58], which were modified using a rigorous selection criterion (FDR ≤0.001 and |log_2_Ratio| ≥1). For each unigene, four FPKM values were generated for each of the four transcriptomes. We used ggplot 2 (http://docs.ggplot2.org/current/geom_point.html) to identify DEGs that play potentially important roles in secondary metabolism [59]. After further investigation, we selected the terpenoid biosynthesis pathways for more detailed analysis. All TF unigenes were annotated in the plantTFDB, and we identified TF genes that showed significant differential expression.

### Construction of protein-protein interaction networks (PPINs) for the terpenoid biosynthesis pathways

Genes that showed differential expression between German and Roman chamomile were aligned to the STRING database (http://string-db.org/) using DIAMOND (https://github.com/bbuchfink/diamond) with parameters “-evalue 1e-5 -outfmt 6 -max-target-seqs 1 –more-sensitive). DEGs with a query coverage ≥50% and an identity ≥40% were identified as homologous interacting proteins and were used to build a protein interaction network. DEGs related to terpenoid biosynthesis pathways were then screened from the interaction networks and these DEGs were examined for enrichment in KEGG pathways (FDR < 0.05). The PPINs were visualized using Cytoscape [41].

### Comparative analyses of German chamomile, Roman chamomile, and other studied species

ORFs from German chamomile and Roman chamomile were clustered using CD-HIT-EST (v4.6.8), respectively. Proteins from *Lactuca sativa* and *Helianthus annuus* were downloaded from NCBI and the longest isoforms were retained for further analysis. Proteins from *Taraxacum kok-saghyz* were downloaded from BIGD (http://bigd.big.ac.cn/search?dbId=gwh&q=PRJCA000437) and proteins from *Chrysanthemum nankingense* were downloaded from the Chrysanthemum Genome Database (http://www.amwayabrc.com/). *Arabidopsis thaliana* protein sequence were downloaded from TAIR (http://www.arabidopsis.org/). The gene families were constructed by orthofinder (v2.2.7) [60]. Briefly, protein sequences from these seven species were used as queries in all-vs-all BLAST searches with an e-value threshold of 1e-5. The BLAST results were then clustered with OrthoMCL [61]. The gene families were visualized by UpSetR (v1.4.0) [62]. Single-copy genes from the seven species were aligned by MAFFT (v7.407) [63]. The phylogenetic tree was constructed by FastTree (2.1.10) [64, 65]. The ORFs from German chamomile, Roman chamomile and proteins from other Compositae family plants were used to predict protein domains based on the Pfam database.

### qRT-PCR verification of selected genes

In order to determine the accuracy of the transcriptome sequencing, qRT-PCR analysis was used to assess the quality of the transcriptomic data. Total RNA was extracted from flowers, and first-strand cDNAs used for qRT-PCR analysis were synthesized from total RNA using a Prime-Script™ 1st Strand cDNA Synthesis Kit (TaKaRa, Dalian, China). Expression of selected terpenoid pathway genes were monitored by qRT-PCR using the SYBR Green qPCR mastermix (Takara, SYBR Premix Ex TaqII™), on a Bio-Rad CFX 96™ real-time PCR system (Bio-Rad), according to the manufacturer’s instructions. Detailed information about the selected unigenes, including their unigene IDs, and all PCR primers sequences are given in Additional file 1. We used the 18S ribosomal RNA gene as an internal reference for gene expression, and the relative expression levels were calculated using the 2^ΔCt^ method [66]. All qRT-PCR analyses were performed using three biological replicates and three technical replicates (means±standard deviation).

## Supplementary information

**Additional file 1.** Essential oil compounds present in disk and ray florets of German chamomile and Roman chamomile.

**Additional file 2.** The enrichment factors and number of genes involved in each pathway derived from comparisons of gene expression in MC_DF-vs-MC_RF, CN_DF-vs-MC_DF, CN_RF-vs-MC_RF and CN_DF-vs-CN_RF. MC_DF stands for German chamomile disk florets, MC_RF for German chamomile ray florets, CN_RF for Roman chamomile ray florets, and CN_DF for Roman chamomile disk florets.

**Additional file 3.** Differentially expressed transcription factor genes in the CN_DF-vs-CN_RF, CN_DF-vs-MC_DF, CN_RF-vs-MC_RF, and MC_DF-vs-MC_RF comparisons.

**Additional file 4.** Information from the PPIN related to the terpenoid biosynthetic pathway in German chamomile and Roman chamomile.

**Additional file 5.** KEGG analysis of unigenes specific to German chamomile and Roman chamomile.

**Additional file 6.** Function of unigenes specific to German chamomile and Roman chamomile.

**Additional file 7.** Transcription levels of genes determined by qRT-PCR.

**Additional file 8: **Supplementary **Figure S1.** COG Functional Classification of German chamomile (A) and Roman chamomile (B). **Figure S2.** KEGG Functional Classification of German chamomile (A) and Roman chamomile (B). **Figure S3**. Validation of candidate unigenes in the German chamomile (A) and Roman chamomile (B) transcriptomes by qRT-PCR. Gene expression levels were determined by qRT-PCR. Transcription levels are indicated as the mean (2^ΔCt^) ± SD. **Figure S4.** Cluster and correlation analyses between German chamomile and Roman chamomile. **Figure S5.** Fully open flowers of German chamomile (A) and Roman chamomile (B) dissected to show the disc and ray florets.

## Data Availability

The Illumina RNA-seq data generated from flowers of *Matricaria recutita* L. and *Chamaemelum nobile* L. are available in the NCBI SRA (http://trace.ncbi.nlm.nih.gov/Traces/sra) under bioproject accession number PRJNA382469 and SRA accession number SRR11011260, SRR11011259, SRR11011258, SRR11011257, SRR11011256 and SRR11011255.

## Abbreviations

AACT: Acetyl-CoA C-acetyltransferase
CMK: 4-diphosphocytidyl-2-C-methyl-D-erythritol kinase
CN: Roman chamomile
COG: Clusters of orthologous groups of proteins
DEGs: Differentially expressed genes
DF: Disk florets
DMAPP: Dimethylallyl diphosphate
DXR: 1-deoxy-D-xylulose-5-phosphate reductoisomerase
DXS: 1-deoxy-D-xylulose-5-phosphate synthase
FDR: False Discovery Rate
FPKM: Fragment Per Kilobase of exon model per Million mapped reads
FPP: Farnesyl-PP
FPPS: Farnesyl diphosphate synthase
GC-MS: Gas Chromatography-Mass Spectrometer
GGPPS: Geranylgeranyl diphosphate synthase
GO: Gene ontology
GPP: geranyl pyrophosphate
GPPS: Geranyl diphosphate synthase
HDR: 4-hydroxy-3-methylbut-2-en-1-yl diphosphate reductase
HDS: 4-hydroxy-3-methylbut-2-en-l-yl diphosphate synthase
HMGR: 3-hydroxy-3-methylglutaryl-coenzyme A reductase
HMGS: Hydroxymethylglutaryl-CoA synthase
IPP: Isopentenylpyrophosphate
IPT: Isopentenyl transferases
KEGG: Kyoto encyclopedia of genes and genomes
MC: German chamomile
MCS: 2-C-methyl-D-erythtitol 2,4-cyclodiphosphate synthase
MEP: Methylerythritol phosphate
MPD: Diphosphomevalonate decarboxylase
MVA: Mevalonate
Nr: Non-redundant protein database
Nt: Non-redundant nucleotide database
PMK: Phosphomevalonate kinase
PPIN: Protein–protein interaction network
PSY: Phytoene synthase
qRT-PCR: Quantitative real-time polymerase chain reaction
RF: Ray florets
SS: Squalene synthase
Swiss-Prot: Annotated protein sequence database
TFs: Transcription factors
TPS: Terpene synthase

## References

[1] Issac O. Recent progress in chamomile research-medicines of plant origin in modern therapy. 1st ed. Czecho-Slovakia: Prague press; 1989. p. 7.

[2] Mann C, Staba EJ (1986). The chemistry, pharmacology, and commercial formulations of chamomile.

[3] Ade-Ademilua O (2009). Tyler’s herbs of Choiceâ’ the therapeutic use of Phytomedicinals by Dennis V. C. Awang. Int J Geogr Inf Syst.

[4] Srivastava JK, Shankar E, Gupta S (2010). Chamomile: a herbal medicine of the past with bright future. Mol Med Rep.

[5] Guenther E (1948). The essential oils: NOSTRAND.

[6] Bassols F, Thomas AF (1991). The occurrence of 3-Phenylpropyl Isobutyrate in Roman Camomile oil. J Essent Oil Res.

[7] Zhao J, Khan SI, Wang M, Vasquez Y, Yang MH, Avula B, Wang YH, Avonto C, Smillie TJ, Khan IA (2014). Octulosonic acid derivatives from Roman chamomile (*Chamaemelum nobile*) with activities against inflammation and metabolic disorder. J Nat Prod.

[8] Piccaglia R, Marotti M, Giovanelli E, Deans SG, Eaglesham E (1993). Antibacterial and antioxidant properties of Mediterranean aromatic plants. Ind Crop Prod.

[9] Lichtenthaler HK (1999). The 1-DEOXY-d-XYLULOSE-5-phosphate pathway of ISOPRENOID biosynthesis in plants. Plant Biol.

[10] Aharoni A, Giri AP, Verstappen FW, Bertea CM, Sevenier R, Sun Z, Jongsma MA, Schwab W, Bouwmeester HJ (2004). Gain and loss of fruit flavor compounds produced by wild and cultivated strawberry species. Plant Cell.

[11] Yamaga Y, Nakanishi K, Fukui H, Tabata M (1993). Intracellular localization of p -hydroxybenzoate geranyltransferase, a key enzyme involved in shikonin biosynthesis. Phytochemistry.

[12] Gutensohn M, Orlova I, Nguyen TTH, Davidovich-Rikanati R, Ferruzzi MG, Sitrit Y, Lewinsohn E, Pichersky E, Dudareva N (2013). Cytosolic monoterpene biosynthesis is supported by plastid-generated geranyl diphosphate substrate in transgenic tomato fruits. Plant J.

[13] Dudareva N, Andersson S, Orlova I, Gatto N, Reichelt M, Rhodes D, Boland W, Gershenzon J (2005). The nonmevalonate pathway supports both monoterpene and sesquiterpene formation in snapdragon flowers. Proc Natl Acad Sci U S A.

[14] Xiang W. The regulation of the metabolism of the upstream genes in the metabolism of terpenoids in tea plants and the preliminary study of the transformation system of tea plant. Hefei: Anhui Agriculture University; 2012.

[15] Gwak Y, Hwang YS, Wang B, Kim M, Jeong J, Lee CG, Hu Q, Han D, Jin E (2014). Comparative analyses of lipidomes and transcriptomes reveal a concerted action of multiple defensive systems against photooxidative stress in *Haematococcus pluvialis*. J Exp Bot.

[16] Herde M, Gärtner K, Köllner TG, Fode B, Boland W, Gershenzon J, Gatz C, Tholl D (2008). Identification and regulation of an Arabidopsis geranyllinalool synthase catalyzing the first step in the formation of the insect-induced volatile C16-homoterpene TMTT involved in indirect plant defense. Plant Cell.

[17] Tai Y, Wei C, Yang H, Zhang L, Chen Q, Deng W, Wei S, Zhang J, Fang C, Ho C (2015). Transcriptomic and phytochemical analysis of the biosynthesis of characteristic constituents in tea ( *Camellia sinensis* ) compared with oil tea ( *Camellia oleifera* ). BMC Plant Biol.

[18] Seema M, Kumar SR, Venkata RDK, Varun D, Shilpashree HB, Shubhra R, Shasany AK, Nagegowda DA (2016). De NovoSequencing and analysis of lemongrass Transcriptome provide first insights into the essential oil biosynthesis of aromatic grasses. Front Plant Sci.

[19] Ishihara KL, Honda MD, Pham DT, Borthakur D (2016). Transcriptome analysis of Leucaena leucocephala and identification of highly expressed genes in roots and shoots.

[20] Wang Z, Gerstein M, Snyder M (2009). RNA-Seq: a revolutionary tool for transcriptomics. Nat Rev Genet.

[21] Cherukupalli N, Divate M, Mittapelli SR, Khareedu VR, Vudem DR (2016). De novoAssembly of leaf Transcriptome in the medicinal PlantAndrographis paniculata. Front Plant Sci.

[22] Gupta P, Goel R, Pathak S, Srivastava A, Singh SP, Sangwan RS, Asif MH, Trivedi PK (2013). De novo assembly, functional annotation and comparative analysis of Withania somnifera leaf and root transcriptomes to identify putative genes involved in the withanolides biosynthesis. PLoS One.

[23] Rajakani R, Narnoliya L, Sangwan NS, Sangwan RS, Gupta V (2014). Subtractive transcriptomes of fruit and leaf reveal differential representation of transcripts in Azadirachta indica. Tree Genet Genomes.

[24] Rastogi S, Meena S, Bhattacharya A, Ghosh S, Shukla RK, Sangwan NS, Lal RK, Gupta MM, Lavania UC, Gupta V (2014). De novo sequencing and comparative analysis of holy and sweet basil transcriptomes. BMC Genomics.

[25] Deng N, Chang E, Li M, Ji J, Yao X, Bartish IV, Liu J, Ma J, Chen L, Jiang Z (2016). Transcriptome Characterization of Gnetum parvifolium Reveals Candidate Genes Involved in Important Secondary Metabolic Pathways of Flavonoids and Stilbenoids. Front Plant Sci.

[26] Lei Yao JH, Chi Q, Chen L (2004). Analysis on the growth habit and essential oil composition of two species of chamomile. Chinese spice fragrance academic seminar 2004.

[27] Mortazavi A, Williams BA, Mccue K, Schaeffer L, Wold B (2008). Mapping and quantifying mammalian transcriptomes by RNA-Seq. Nat Methods.

[28] Tai Y, Ling C, Wang C, Wang H, Su L, Yang L, Jiang W, Yu X, Zheng L, Feng Z, et al. Analysis of terpenoid biosynthesis pathways in German chamomile (*Matricaria recutita*) and Roman chamomile (*Chamaemelum nobile*) based on co-expression networks. Genomics. 2020;112(2):1055–64. 10.1016/j.ygeno.2019.10.023 31706023

[29] Tong Y, Su P, Zhao Y, Zhang M, Wang X, Liu Y, Zhang X, Gao W, Huang L (2015). Molecular cloning and characterization of DXS and DXR genes in the Terpenoid biosynthetic pathway of *Tripterygium wilfordii*. Int J Mol Sci.

[30] Xu C, Li H, Yang X, Gu C, Mu H, Yue Y, Wang L (2016). Cloning and expression analysis of MEP pathway enzyme-encoding genes in Osmanthus fragrans. Genes.

[31] Cordoba E, Porta H, Arroyo A, San RC, Medina L, Rodríguez-Concepción M, León P (2011). Functional characterization of the three genes encoding 1-deoxy-D-xylulose 5-phosphate synthase in maize. J Exp Bot.

[32] Kim SM, Kuzuyama T, Kobayashi A, Sando T, Chang YJ, Kim SU (2008). 1-Hydroxy-2-methyl-2-( E )-butenyl 4-diphosphate reductase (IDS) is encoded by multicopy genes in gymnosperms *Ginkgo biloba* and *Pinus taeda*. Planta.

[33] Tholl D (2006). Terpene synthases and the regulation, diversity and biological roles of terpene metabolism. Curr Opin Plant Biol.

[34] Vom ED, Kijne JW, Memelink J (2002). Transcription factors controlling plant secondary metabolism: what regulates the regulators?. Phytochemistry.

[35] Harborne JB (1996). Herbal medicines: A guide for health-care professionals: by CA Newall, LA Anderson and JD Phillipson, The Pharmaceutical Press, London, 1996, 296 pp., £30.00. ISBN 0-85369-289-0. Phytochemistry.

[36] Foster S (1998). The complete German commission E monographs: therapeutic guide to herbal medicines (book review). J Altern Complement Med.

[37] Singh O, Khanam Z, Misra N, Srivastava MK (2011). Chamomile (*Matricaria chamomilla* L.): An overview. Pharmacogn Rev.

[38] Omidbaigi R, Sefidkon F, Kazemi F (2004). Influence of drying methods on the essential oil content and composition of Roman chamomile. Flavour Fragrance J.

[39] Brendler T. Chamomile. Industrial Profiles. Economic Botany. 2006;60(2):197.

[40] Zeng X, Cai L, Zheng R, Xuan C, Jing L, Zou J, Wang C (2016). Emission and Accumulation of Monoterpene and the Key Terpene Synthase (TPS) Associated with Monoterpene Biosynthesis In*osmanthus fragrans*lour. Front Plant Sci.

[41] Shannon P, Markiel A, Ozier O, Baliga NS, Wang JT, Ramage D, Amin N, Schwikowski B, Ideker T (2003). Cytoscape: a software environment for integrated models of biomolecular interaction networks. Genome Res.

[42] Xie T, Wang S, Huang L, Wang X, Kang Lp, Guo L-p. [Transcriptome-based bioinformatics analysis of Arnebia euchroma ERF transcription factor family]. Zhongguo Zhong Yao Za Zhi. 2014;39(24):4732–9. 25898569

[43] Yang L, Ding G, Lin H, Cheng H, Kong Y, Wei Y, Fang X, Liu R, Wang L, Chen X (2013). Transcriptome analysis of medicinal plant Salvia miltiorrhiza and identification of genes related to Tanshinone biosynthesis. PLoS One.

[44] Razboršek MI, Vončina DB, Doleček V, Vončina E (2008). Determination of Oleanolic, Betulinic and Ursolic acid in Lamiaceae and mass spectral fragmentation of their Trimethylsilylated derivatives. Chromatographia.

[45] Spyropoulou EA, Haring MA, Schuurink RC (2014). RNA sequencing on Solanum lycopersicum trichomes identifies transcription factors that activate terpene synthase promoters. BMC Genomics.

[46] Suttipanta N, Pattanaik S, Kulshrestha M, Patra B, Singh SK, Yuan L (2011). The transcription factor CrWRKY1 positively regulates the terpenoid indole alkaloid biosynthesis in Catharanthus roseus. Plant Physiol.

[47] Li J, Blue R, Zeitler B, Strange TL, Pearl JR, Huizinga DH, Evans S, Gregory PD, Urnov FD, Petolino JF (2013). Activation domains for controlling plant gene expression using designed transcription factors. Plant Biotechnol J.

[48] Banerjee A, Hamberger B (2018). P450s controlling metabolic bifurcations in plant terpene specialized metabolism. Phytochem Rev.

[49] Zhao YJ, Cheng QQ, Su P, Chen X, Wang XJ, Gao W, Huang LQ (2014). Research progress relating to the role of cytochrome P450 in the biosynthesis of terpenoids in medicinal plants. Appl Microbiol Biotechnol.

[50] Salamon I. Chamomile biodiversity of the essential oil qualitative-quantitative characteristics. In: Innovations in chemical biology. Edited by Şener B. Dordrecht: Springer Netherlands; 2009. p. 83–90.

[51] Bohn RB, Messina J, Liu F, Tong J, Mao J (2011). NIST cloud computing reference architecture. IEEE World Congress on Services.

[52] Chengying WXS. Method for high-quality total RNA isolation from tea plant [*Camellia sinensis*] (L.) O.Kuntze. Hefei: Journal of Anhui Agricultural University; 2007.

[53] Grabherr MG, Haas BJ, Yassour M, Levin JZ, Thompson DA, Amit I, Adiconis X, Fan L, Raychowdhury R, Zeng Q (2011). Full-length transcriptome assembly from RNA-Seq data without a reference genome. Nat Biotechnol.

[54] Tatusov RL, Natale DA, Garkavtsev IV, Tatusova TA, Shankavaram UT, Rao BS, Kiryutin B, Galperin MY, Fedorova ND, Koonin EV (2001). The COG database: new developments in phylogenetic classification of proteins from complete genomes. Nucleic Acids Res.

[55] Kanehisa M, Goto S, Kawashima S, Okuno Y, Hattori M (2004). The KEGG resource for deciphering the genome. Nucleic Acids Res.

[56] Ye J, Fang L, Zheng H, Zhang Y, Chen J, Zhang Z, Wang J, Li S, Li R, Bolund L (2006). WEGO: a web tool for plotting GO annotations. Nucleic Acids Res.

[57] Conesa A, Götz S, Garcíagómez JM, Terol J, Talón M, Robles M (2005). Blast2GO: a universal tool for annotation, visualization and analysis in functional genomics research. Bioinformatics.

[58] Audic S, Claverie JM (1997). The significance of digital gene expression profiles. Genome Res.

[59] Trapnell C, Hendrickson DG, Sauvageau M, Goff L, Rinn JL, Pachter L (2012). Differential analysis of gene regulation at transcript resolution with RNA-seq. Nat Biotechnol.

[60] Emms DM, Kelly S (2015). OrthoFinder: solving fundamental biases in whole genome comparisons dramatically improves orthogroup inference accuracy. Genome Biol.

[61] Li L, Stoeckert CJ, Roos DS (2003). OrthoMCL: identification of ortholog groups for eukaryotic genomes. Genome Res.

[62] Conway JR, Lex A, Gehlenborg N (2017). UpSetR: an R package for the visualization of intersecting sets and their properties. Bioinformatics.

[63] Kazutaka K, Standley DM (2013). MAFFT multiple sequence alignment software version 7: improvements in performance and usability. Mol Biol Evol.

[64] Price MN, Dehal PS, Arkin AP (2010). FastTree 2--approximately maximum-likelihood trees for large alignments. PLoS One.

[65] Price MN, Dehal PS, Arkin AP (2009). FastTree: computing large minimum evolution trees with profiles instead of a distance matrix. Mol Biol Evol.

[66] Livak KJ, Schmittgen TD (2001). Analysis of relative gene expression data using real-time quantitative PCR and the 2 (−Delta Delta C (T)) method. Methods.

