# Supplementary material for: Phytochemical and comparative transcriptome analyses reveal different regulatory mechanisms in the terpenoid biosynthesis pathways between Matricaria recutita L. and Chamaemelum nobile L
Source: BMC Genomics. 2020 Feb 18;21:169. doi: 10.1186/s12864-020-6579-z (PMC7029581; doi:10.1186/s12864-020-6579-z)
Supplement: Supplementary file 1 — Additional file 1. Essential oil compounds present in disk and ray florets of German chamomile and Roman chamomile. [file 12864_2020_6579_MOESM1_ESM.docx]

Essential oil compounds present in disk and ray florets of German chamomile and Roman chamomile

| **Code** | | Compounds | Molecular formula | RT (min) | CAS | Relative area percent (%)（mean） | | | |
| --- | --- | --- | --- | --- | --- | --- | --- | --- | --- |
|  |  |  |  |  |  | MC-DF | MC-RF | CN-DF | CN-RF |
| 1 | Propanoic acid, 2-methyl-, 2-methylpropyl ester | | C_8_H_16_O_2_ | 9.762 | 97-85-8 | /aA | /aA | 0.794±0.030bB | /aA |
| 2 | Isobutyl methacrylate | | C_8_H_14_O_2_ | 10.427 | 97-86-9 | /aA | /aA | 1.097±0.163bB | /aA |
| 3 | (+)-alpha-pinene | | C_10_H_16_ | 10.544 | 7785-70-8 | /aA | /aA | 1.819±0.258bB | /aA |
| 4 | 3-Methyl,2-butenoic acid, isopropyl ester | | C_8_H_14_O_2_ | 11.906 | 25859-51-2 | /aA | /aA | 0.947±0.153bB | /aA |
| 5 | Butanoic acid, 2-methyl-, 2-methylpropyl ester | | C_9_H_18_O_2_ | 12.218 | 2445-67-2 | /aA | /aA | 1.036±0.172bB | /aA |
| 6 | Propanoic acid, 2-methyl-, 2-methylbutyl ester | | C_9_H_18_O_2_ | 12.575 | 2445-69-4 | /aA | /aA | 2.672±0.46bB | /aA |
| 7 | Cyclopropanecarboxylic acid,3-methylbutyl ester | | C_9_H_16_O_2_ | 13.335 | 1000245-65-3 | /aA | /aA | 3.916±0.625bB | /aA |
| 8 | 3-Methylbut-2-enoic acid, 2-methylpentyl ester | | C_11_H_20_O_2_ | 13.577 | 1000355-12-8 | /aA | /aA | 14.277±2.348bB | 2.615±0.464aA |
| 9 | Butanoic acid, 3-methylbut-2-enyl ester | | C_9_H_16_O_2_ | 13.696 | 1000299-11-8 | /aA | /aA | 3.221±0.55bB | /aA |
| 10 | 3-Methylbut-2-enoic acid, 4-nitrophenyl ester | | C_11_H_11_NO_4_ | 13.954 | 1000307-59-8 | /aA | /aA | 14.085±2.394bB | /aA |
| 11 | Cyclopropanecarboxylic acid, 3-methylbut-2-enyl ester | | C_9_H_14_O_2_ | 14.502 | 1000299-37-4 | /aA | /aA | 2.99±0.517bB | /aA |
| 12 | Butanoic acid, 2-methyl-, 2-methylbutyl ester | | C_10_H_20_O_2_ | 15.105 | 2445-78-5 | /aA | /aA | 3.121±0.44bB | /aA |
| 13 | Hexanoic acid, 2-methylpropyl ester | | C_10_H_20_O_2_ | 15.273 | 105-79-3 | /aA | /aA | 0.842±0.185bB | /aA |
| 14 | Propanoic acid, 2-methyl-, hexyl ester | | C_10_H_20_O_2_ | 15.438 | 2349-07-7 | /aA | /aA | 3.353±0.599bB | /aA |
| 15 | .alpha.-Campholenal | | C_10_H_16_O | 16.022 | 4501-58-0 | /aA | /aA | 0.81±0.16bB | /aA |
| 16 | Valeric acid, 3-methylbut-2-enyl ester | | C_10_H_18_O_2_ | 16.203 | 1000292-48-5 | /aA | /aA | 1.86±0.34bB | /aA |
| 17 | Cyclopropanecarboxylic acid, 2-methylpentyl ester | | C_10_H_18_O_2_ | 16.285 | 1000354-65-8 | /aA | /aA | 7.375±1.127bB | /aA |
| 18 | 3-Methylbut-2-enoic acid, 2-methylbutyl ester | | C_10_H_18_O_2_ | 16.357 | 1000331-14-7 | /aA | /aA | 15.207±2.56bB | 0.819±0.143aA |
| 19 | (+)-cis-sabinol | | C_10_H_16_O | 16.601 | 471-16-9 | /aA | /aA | /aA | 4.11±0.462bB |
| 20 | Pinocarvone | | C_10_H_14_O | 17.17 | 30460-92-5 | /aA | /aA | 16.993±3.035bB | 2.220±0.396aA |
| 21 | Prenyl senecioate | | C_10_H_16_O_2_ | 17.531 | 72779-06-7 | /aA | /aA | 18.771±3.448bB | 0.694±0.126aA |
| 22 | 4-Methylpentan-2-yl 2-methylbutanoate | | C_11_H_22_O_2_ | 17.902 | 67883-79-8 | /aA | /aA | 3.021±0.557bB | /aA |
| 23 | .alpha.-Terpineol | | C_10_H_18_O | 17.953 | 10482-56-1 | /aA | /aA | 0.961±0.083bB | /aA |
| 24 | Myrtenal | | C_10_H_14_O | 18.048 | 564-94-3 | /aA | /aA | 4.274±3.368aA | /aA |
| 25 | (-)-Myrtenol | | C_10_H_16_O | 18.183 | 19894-97-4 | /aA | /aA | /aA | 1.327±0.278bB |
| 26 | 3,7-Nonadien-2-one, 4,8-dimethyl- | | C_11_H_18_O | 18.737 | 817-88-9 | 1.692±0.031aA | /bB | /bB | /bB |
| 27 | 2-Hydroxy-2-methyl-but-3-enyl 2-methyl-2(Z)-butenoate | | C_10_H_16_O_3_ | 18.965 | 80758-67-4 | /aA | /aA | 6.853±1.404bB | /aA |
| 28 | Nonanoic acid | | C_9_H_18_O_2_ | 19.577 | 112-05-0 | /aA | /aA | 2.967±0.537bB | 1.010±0.189cC |
| 29 | 4,8-Dimethyl-nona-3,8-dien-2-one | | C_11_H_18_O | 19.73 | 1000190-70-5 | 3.514±0.024aA | 0.976±0.005bB | /cC | /cC |
| 30 | (Z)-Hex-3-enyl (E)-2-methylbut-2-enoate | | C_11_H_18_O_2_ | 19.831 | 1000373-73-0 | /aA | /aA | 2.111±0.377bB | /aA |
| 31 | Isopentyl 3-hydroxy-2-methylenebutanoate | | C_10_H_18_O_3_ | 20.05 | 80758-69-6 | /aA | /aA | 1.832±0.410bB | /aA |
| 32 | 2,4-Decadienal | | C_10_H_16_O | 21.036 | 2363-88-4 | /aA | /aA | /aA | 0.767±0.141bB |
| 33 | Eugenol | | C_10_H_12_O_2_ | 21.947 | 97-53-0 | /aA | /aA | 0.882±0.170bB | /aA |
| 34 | .alpha.-Cubebene | | C_15_H_24_ | 22.711 | 17699-14-8 | /aA | /aA | 3.324±0.309bB | /aA |
| 35 | β-elemene | | C_15_H_24_ | 22.961 | 515-13-9 | /aA | /aA | 1.087±0.162bB | /aA |
| 36 | β-cubebene | | C_15_H_24_ | 23.815 | 13744-15-5 | /aA | /aA | 1.894±0.172bB | /aA |
| 37 | Caryophyllene | | C_15_H_24_ | 23.91 | 87-44-5 | /aA | /aA | 2.914±0.555bB | /aA |
| 38 | (E)-.beta.-Famesene | | C_15_H_24_ | 24.311 | 18794-84-8 | 14.466±0.128aA | 2.987±0.037bB | 4.196±0.782bB | 8.529±1.48cC |
| 39 | .gamma.-Muurolene | | C_15_H_24_ | 25.23 | 30021-74-0 | /aA | /aA | /aA | 0.451±0.069bB |
| 40 | (+)-γ-Cadinene | | C_15_H_24_ | 25.254 | 39029-41-9 | /aA | /aA | 3.593±0.583bB | /aA |
| 41 | (+)-germacrene-D | | C_15_H_24_ | 25.533 | 23986-74-5 | 2.294±0.029aA | /aA | 22.38±3.808bB | 1.374±0.237aA |
| 42 | Isolongifolene, 4,5-dehydro- | | C_15_H_22_ | 25.723 | 1000152-07-1 | /aA | /aA | 11.182±1.964bB | 0.578±0.124aA |
| 43 | 2-Naphthyl methyl ketone | | C_12_H_10_O | 25.767 | 93-08-3 | /aA | /aA | 1.014±0.246bB | 0.755±0.138cC |
| 44 | (+)-β-selinene | | C_15_H_24_ | 25.804 | 17066-67-0 | /aA | /aA | 1.137±0.124bB | 0.568±0.107cC |
| 45 | .alpha.-Muurolene | | C_15_H_24_ | 25.885 | 31983-22-9 | /aA | /aA | 1.78±0.310bB | /aA |
| 46 | Tetradecanal | | C_14_H_28_O | 25.908 | 124-25-4 | /aA | /aA | /aA | 0.565±0.049aA |
| 47 | Elixene | | C_15_H_24_ | 25.944 | 3242-08-8 | 1.413±0.006aA | /bB | 0.807±0.066cC | /bB |
| 48 | (+)-δ-cadinene | | C_15_H_24_ | 26.457 | 483-76-1 | /aA | /aA | 5.972±1.07bB | 0.646±0.102aA |
| 49 | Dodecanoic acid | | C_12_H_24_O_2_ | 27.409 | 143-07-7 | /aA | 2.214±0.086bBC | 2.58±0.437bB | 1.524±0.294cC |
| 50 | Nerolidol 2 | | C_15_H_26_O | 27.571 | 1000285-43-6 | 11.589±0.213aA | 6.587±0.051bB | /cC | 1.837±0.315dD |
| 51 | Naphthalene, 2,6-dimethyl- | | C_12_H_12_ | 27.91 | 581-42-0 | /aA | /aA | 1.630±0.320bB | /aA |
| 52 | Espatulenol | | C_15_H_24_O | 28.632 | 6750-60-3 | 27.357±0.272aA | 7.765±0.142bB | 2.589±0.623cC | 3.965±0.701dD |
| 53 | Caryophyllene oxide | | C_15_H_24_O | 28.828 | 1139-30-6 | 1.309±0.04aA | /bB | /bB | /bB |
| 54 | 1H-Inden-1-one, 2,3,3a,4,5,7a-hexahydro-4,4,7a-trimethyl- | | C_12_H_18_O | 29.736 | 109629-65-4 | 9.000±0.071aA | /bB | /bB | /bB |
| 55 | .tau.-Cadinol | | C_15_H_26_O | 30.498 | 5937-11-1 | /aA | /aA | 1.037±0.318bB | /aA |
| 56 | Tridecanoic acid | | C_13_H_26_O_2_ | 30.502 | 638-53-9 | /aA | /aA | /aA | 0.552±0.08bB |
| 57 | .tau.-Muurolol | | C_15_H_26_O | 30.573 | 19912-62-0 | /aA | /aA | 1.589±0.294bB | /aA |
| 58 | .alpha.-Cadinol | | C_15_H_26_O | 30.96 | 481-34-5 | /aA | /aA | 3.53±0.668bB | /aA |
| 59 | α-Bisabolol oxide B | | C_15_H_26_O_2_ | 31.051 | 26184-88-3 | 62.190±24.702aA | 40.269±0.597aAB | 2.202±0.384bB | 13.234±2.325bB |
| 60 | Selina-6-en-4-ol | | C_15_H_26_O | 31.174 | 1000140-23-2 | /aA | /aA | 1.270±0.242bB | /aA |
| 61 | Humulane-1,6-dien-3-ol | | C_15_H_26_O | 31.457 | 1000140-23-1 | /aA | /aA | 2.060±0.497bB | /aA |
| 62 | α-Bisabolol | | C_15_H_26_O | 31.906 | 515-69-5 | 25.033±5.681aA | 5.147±0.794bB | /bB | 0.560±0.097bB |
| 63 | Pentadecanal- | | C_15_H_30_O | 32.33 | 2765-11-9 | /aA | /aA | 2.196±0.409bB | 0.747±0.274cC |
| 64 | Chamazulene | | C_14_H_16_ | 33.589 | 529-05-5 | 64.019±0.625aA | 34.601±1.733bB | 5.613±1.074cC | 9.796±1.729dD |
| 65 | Tetradecanoic acid | | C_14_H_28_O_2_ | 33.854 | 544-63-8 | 8.381±0.199aA | 15±0.983bB | 9.899±1.639aA | 10.01±1.766aA |
| 66 | α-Bisabolol oxide A | | C_15_H_26_O_2_ | 34.032 | 22567-36-8 | 105.813±18.649aA | 52.926±8.73bB | 2.150±0.091cC | 7.935±1.496cC |
| 67 | 4,4'-Dimethylbiphenyl | | C_14_H_14_ | 34.926 | 613-33-2 | 1.304±0.019aA | /bB | 1.609±0.386aA | 0.781±0.203cB |
| 68 | Corymbolone | | C_15_H_24_O_2_ | 35.114 | 97094-19-4 | /aA | /aA | 6.649±1.731bB | 1.363±0.147aA |
| 69 | Phytone | | C_18_H_36_O | 36.056 | 502-69-2 | /aA | 2.004±0.028bA | 7.413±1.467cB | 5.071±0.861dA |
| 70 | Oxacyclotetradecane-2,11-dione, 13-methyl- | | C_14_H_24_O_3_ | 36.119 | 74685-36-2 | /aA | /aA | 1.042±0.183bB | /aA |
| 71 | 14-Pentadecenoic acid | | C_15_H_28_O_2_ | 36.121 | 17351-34-7 | /aA | 2.611±0.03bB | /aA | /aA |
| 72 | Pentadecanoic acid | | C_15_H_30_O_2_ | 36.644 | 1002-84-2 | /aA | 11.174±0.319bB | 7.027±1.232cC | 6.783±1.532cC |
| 73 | Phthalic acid, butyl 2-pentyl ester | | C_17_H_24_O_4_ | 36.66 | 1000315-47-6 | /aA | /aA | /aA | 0.896±0.16bB |
| 74 | Tonghaosu | | C_13_H_12_O_2_ | 37.851 | 16863-61-9 | 1.444±0.021aA | /bB | /bB | /bB |
| 75 | Heptadecanal | | C_17_H_34_O | 38.271 | 1000376-70-0 | /aA | /aA | 1.488±0.283bB | /aA |
| 76 | Hexadecanoic acid, methyl ester | | C_17_H_34_O_2_ | 38.357 | 112-39-0 | /aA | 2.140±0.037bAC | 7.272±1.307cB | 3.628±0.942bC |
| 77 | Hexadecenoic acid, Z-11- | | C_16_H_30_O_2_ | 38.841 | 2416-20-8 | /aA | 6.838±0.406bA | /aA | 2.518±0.21abA |
| 78 | Dibutyl phthalate | | C_16_H_22_O_4_ | 39.278 | 84-74-2 | /aA | /aA | /aA | 0.917±0.157bB |
| 79 | n-Hexadecanoic acid | | C_16_H_32_O_2_ | 39.47 | 57-10-3 | 23.158±0.077aA | /aA | 81.229±5.281bB | 34.407±29.346aA |
| 80 | Azulen-2-ol, 1,4-dimethyl-7-(1-methylethyl)- | | C_15_H_18_O | 39.582 | 18937-66-1 | 4.582±0.067aA | /bB | /bB | /bB |
| 81 | margaric acid | | C_17_H_34_O_2_ | 41.972 | 506-12-7 | /aA | /aA | /aA | 0.720±0.166bB |
| 82 | 9,12-Octadecadienoic acid, methyl ester | | C_19_H_34_O_2_ | 42.749 | 2462-85-3 | /abA | 1.747±0.061acA | 7.436±1.570dB | 1.794±0.329aA |
| 83 | Linoleoyl Chloride | | C_18_H_31_ClO | 42.918 | 7459-33-8 | /aA | /aA | 4.482±0.824bB | /aA |
| 84 | Methyl stearate | | C_19_H_38_O_2_ | 43.511 | 112-61-8 | /aA | /aA | 1.479±0.472bB | /aA |
| 85 | linoleic acid | | C_18_H_32_O_2_ | 43.761 | 60-33-3 | 9.793±0.178aAB | /aB | 36.262±21.878bAC | 52.387±4.089bC |
| 86 | 17-Octadecynoic acid | | C_18_H_32_O_2_ | 43.905 | 34450-18-5 | 4.631±1.605aA | /bB | /bB | /bB |
| 87 | α-linolenic acid | | C_18_H_30_O_2_ | 43.906 | 463-40-1 | /aA | /aA | /aA | 9.626±2.214bB |
| 88 | Methyl Linolenate | | C_19_H_32_O_2_ | 43.912 | 301-00-8 | /aA | 1.251±0.121bB | /aA | 1.011±0.156cB |
| 89 | 9,17-Octadecadienal, (Z)- | | C_18_H_32_O | 44.089 | 56554-35-9 | /aA | /aA | 17.881±2.122bB | /aA |
| 90 | Cyclooctene, 5,6-diethenyl-, trans- | | C_12_H_18_ | 44.244 | 53264-71-4 | /aA | /aA | 1.013±0.24bB | /aA |
| 91 | Z,E-7,11-Hexadecadien-1-yl acetate | | C_18_H_32_O_2_ | 44.366 | 51607-94-4 | /aA | 4.376±0.098bB | /aA | /aA |
| 92 | Octadecanoic acid | | C_18_H_36_O_2_ | 44.469 | 57-11-4 | /aA | 1.554±0.222bB | 2.366±0.529bB | 1.787±0.509bB |
| 93 | Tetracosane | | C_24_H_50_ | 51.646 | 646-31-1 | 6.597±1.308aA | 4.203±0.063aA | 15.994±12.166aA | 2.546±0.444aA |
