# Supplementary material for: Phytochemical and comparative transcriptome analyses reveal different regulatory mechanisms in the terpenoid biosynthesis pathways between Matricaria recutita L. and Chamaemelum nobile L
Source: BMC Genomics. 2020 Feb 18;21:169. doi: 10.1186/s12864-020-6579-z (PMC7029581; doi:10.1186/s12864-020-6579-z)
Supplement: Supplementary file 8 — Additional file 8: Supplementary Figure S1. COG Functional Classification of German chamomile (A) and Roman chamomile (B). Figure S2. KEGG Functional Classification of German chamomile (A) and Roman chamomile (B). Figure S3. Validation of candidate unigenes in the German chamomile (A) and Roman chamomile (B) transcriptomes by qRT-PCR. Gene expression levels were determined by qRT-PCR. Transcription levels are indicated as the mean (2ΔCt) ± SD. Figure S4. Cluster and correlation analyses between German chamomile and Roman chamomile. Figure S5. Fully open flowers of German chamomile (A) and Roman chamomile (B) dissected to show the disc and ray florets. [file 12864_2020_6579_MOESM8_ESM.ppt]

## Slide 1
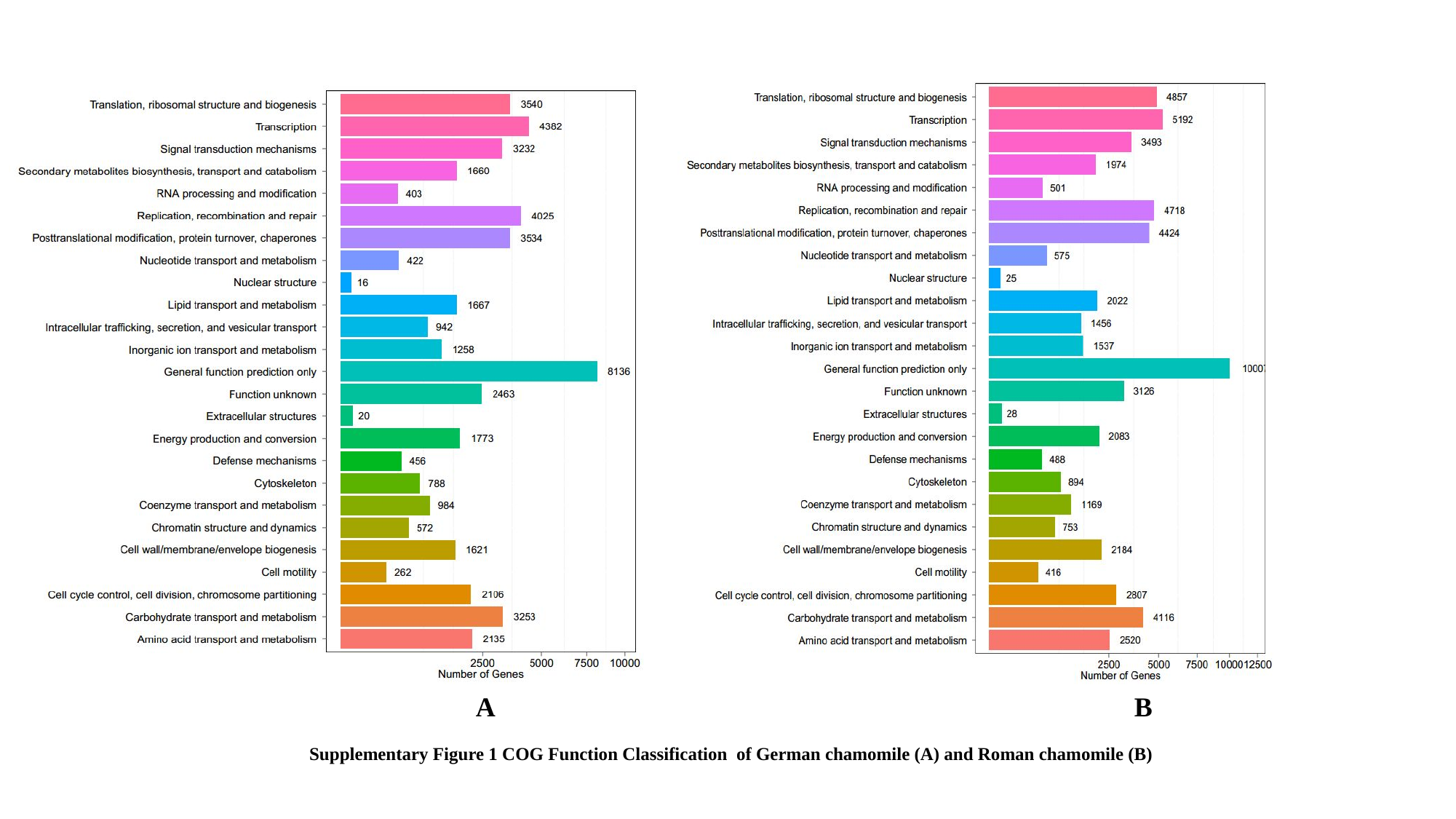

A
B
Supplementary Figure 1 COG Function Classification of German chamomile (A) and Roman chamomile (B)

## Slide 2
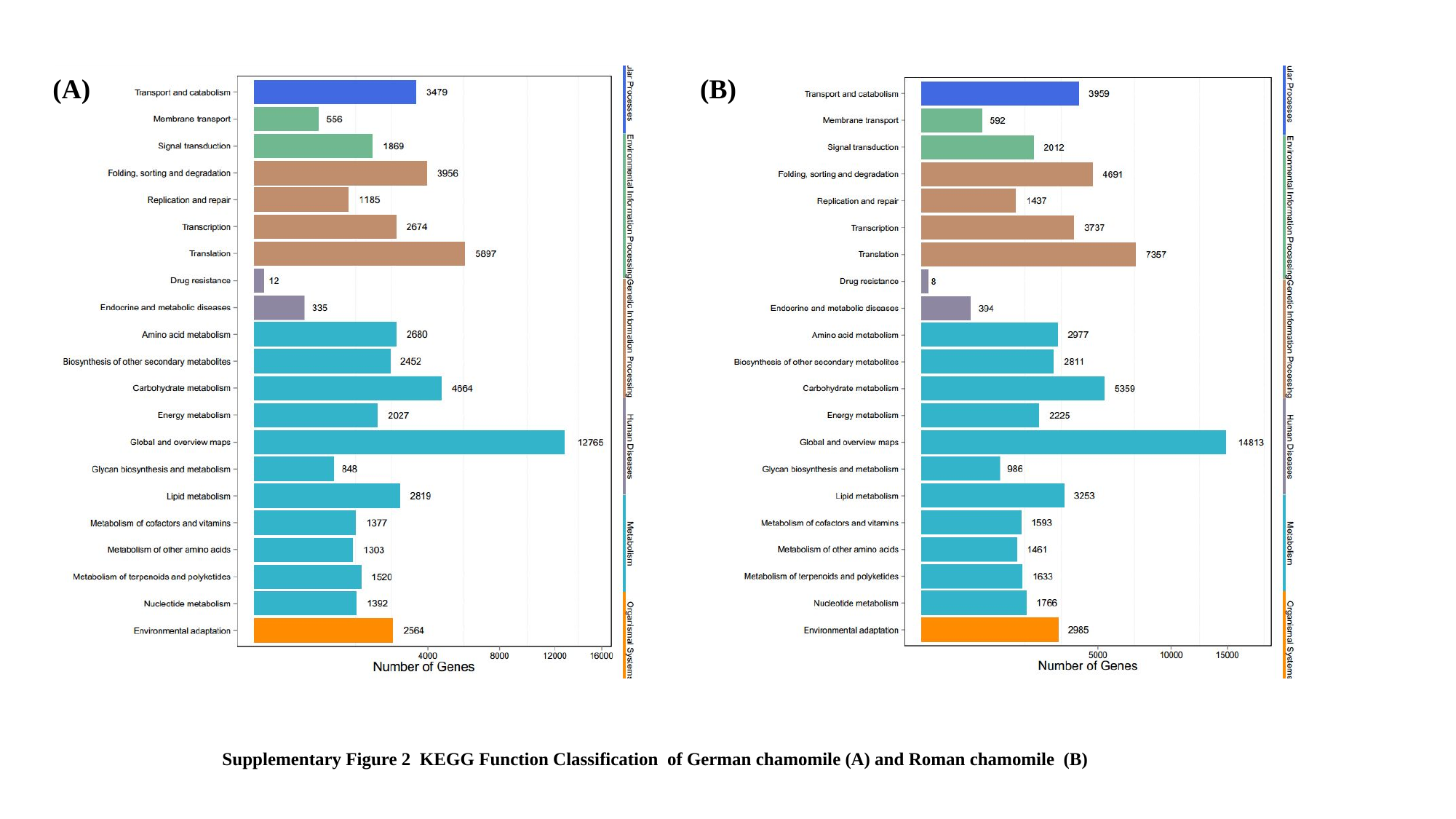

(A)
(B)
Supplementary Figure 2 KEGG Function Classification of German chamomile (A) and Roman chamomile (B)

## Slide 3
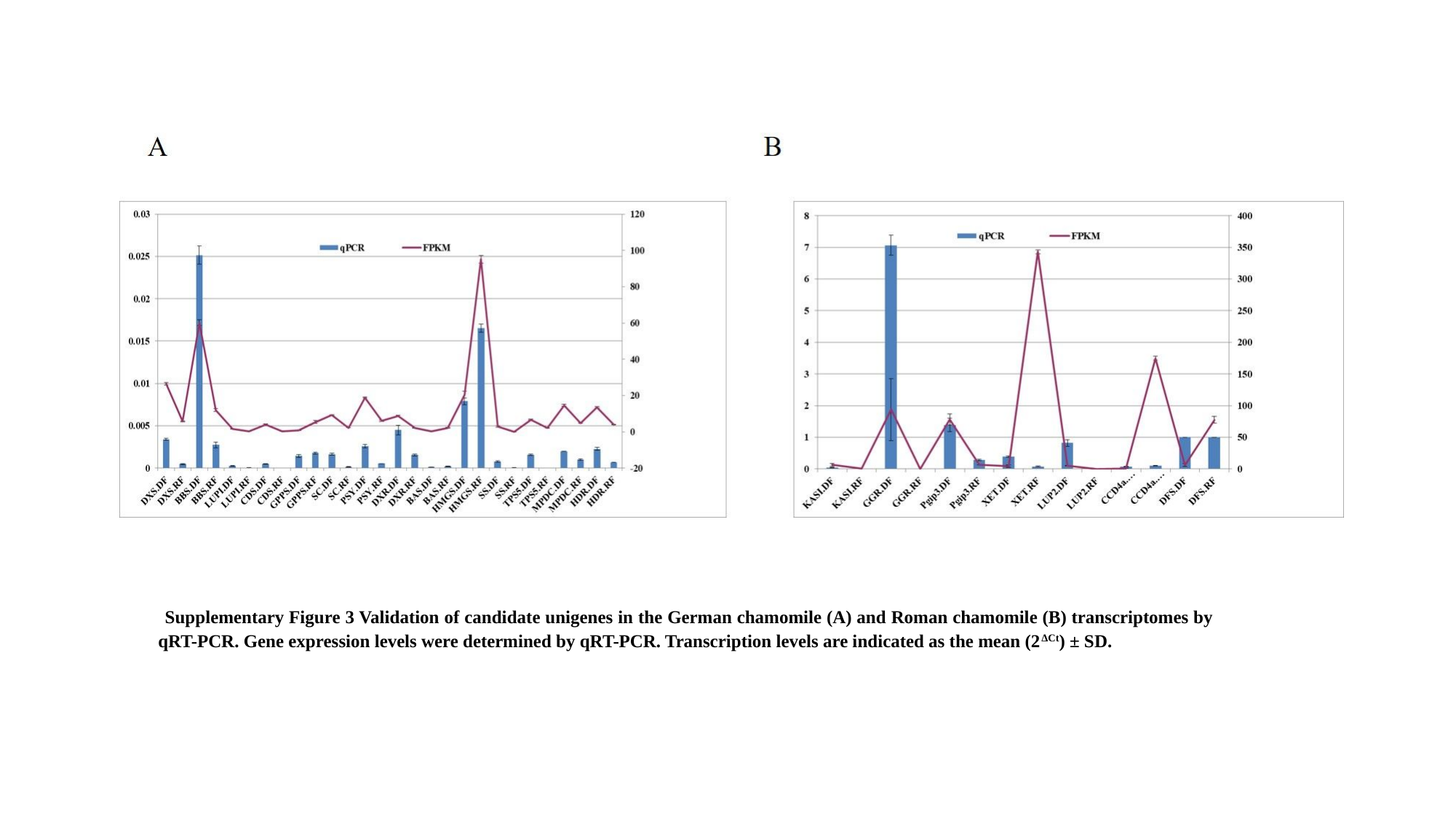

Supplementary Figure 3 Validation of candidate unigenes in the German chamomile (A) and Roman chamomile (B) transcriptomes by qRT-PCR. Gene expression levels were determined by qRT-PCR. Transcription levels are indicated as the mean (2ΔCt) ± SD.

## Slide 4
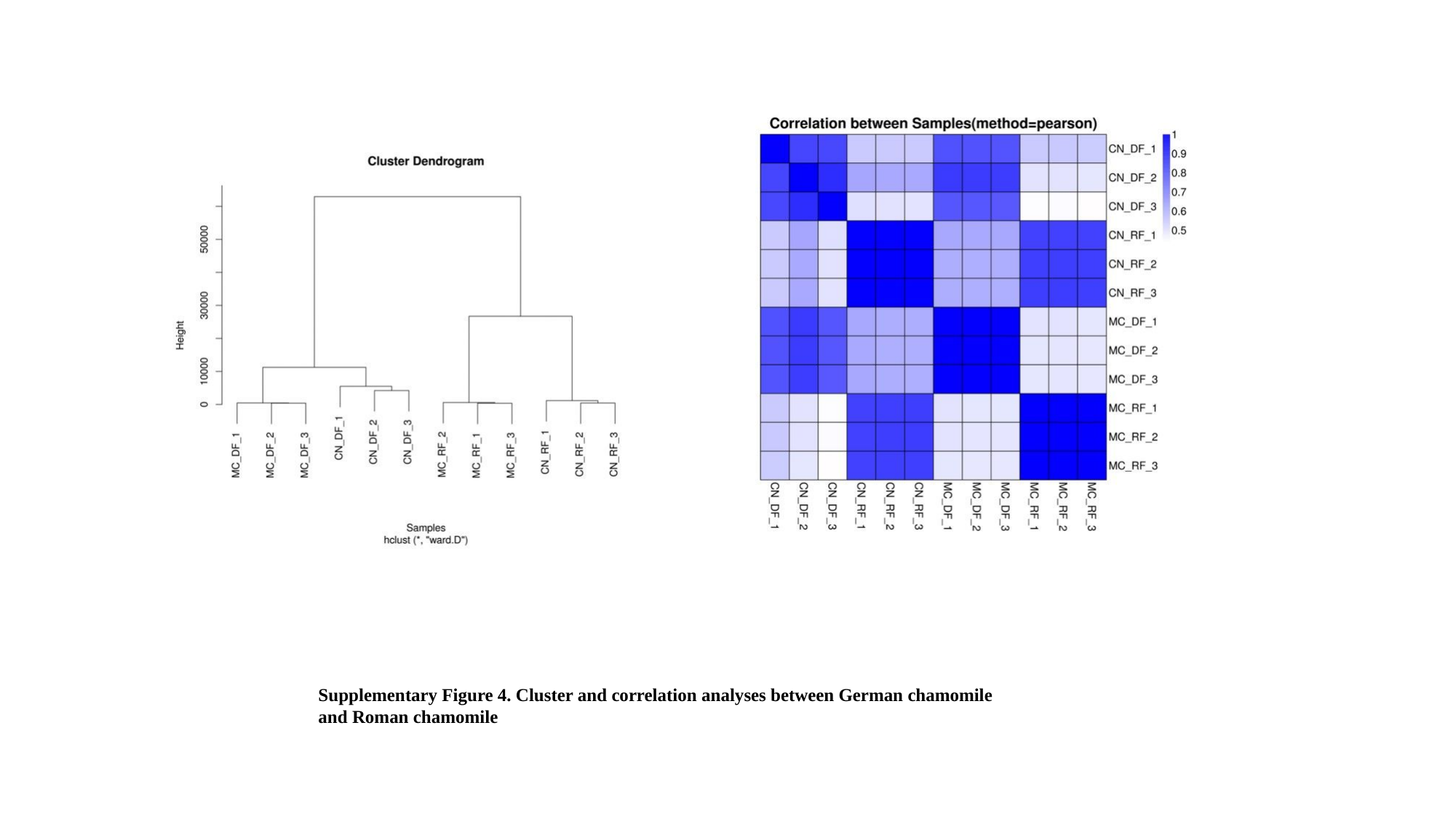

Supplementary Figure 4. Cluster and correlation analyses between German chamomile and Roman chamomile

## Slide 5
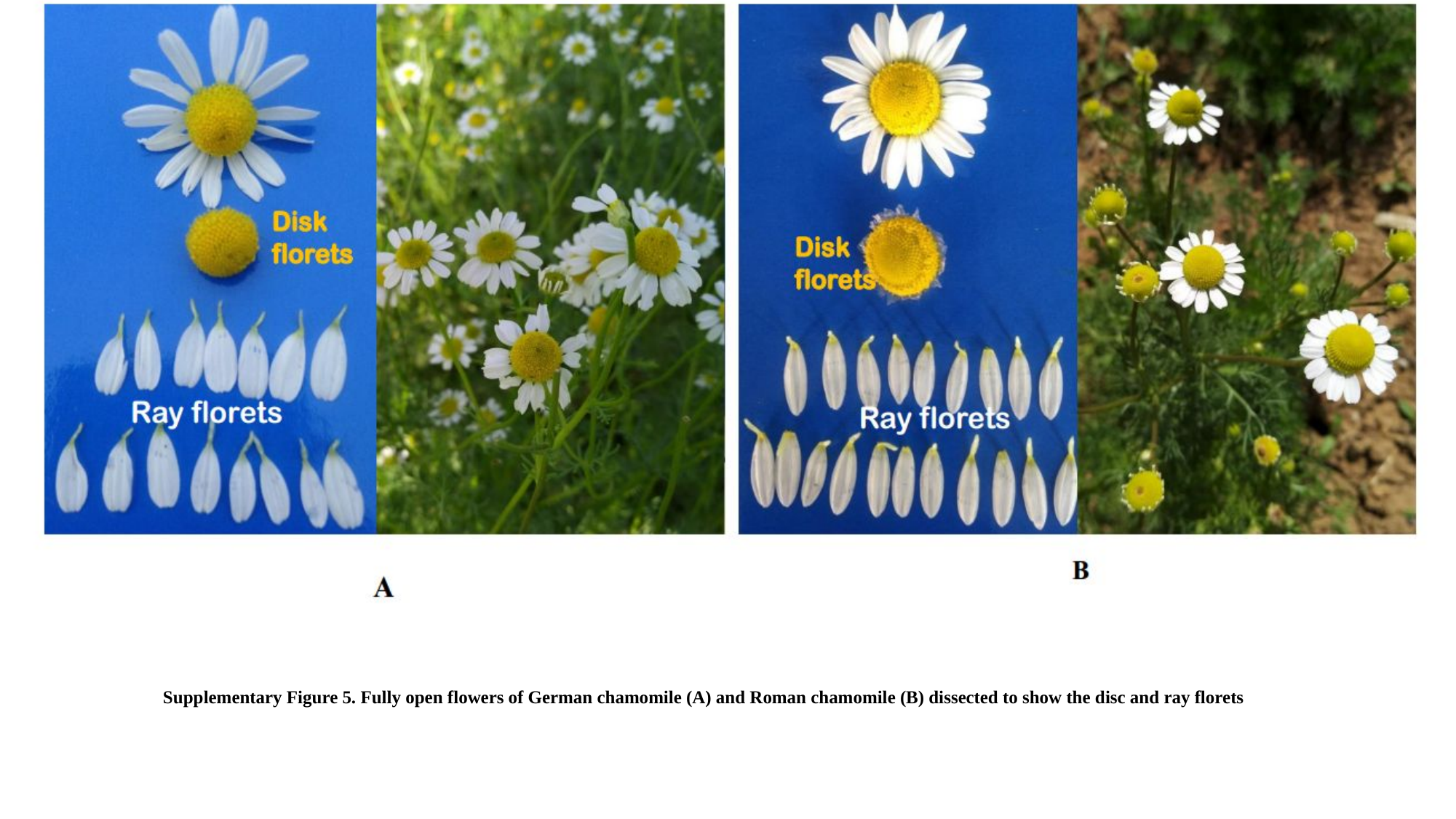

Supplementary Figure 5. Fully open flowers of German chamomile (A) and Roman chamomile (B) dissected to show the disc and ray florets
